# Biofilm Microenvironment-Responsive Nanotheranostics for Dual-Mode Imaging and Hypoxia-Relief-Enhanced Photodynamic Therapy of Bacterial Infections

**DOI:** 10.34133/2020/9426453

**Published:** 2020-04-12

**Authors:** Weijun Xiu, Siyu Gan, Qirui Wen, Qiu Qiu, Sulai Dai, Heng Dong, Qiang Li, Lihui Yuwen, Lixing Weng, Zhaogang Teng, Yongbin Mou, Lianhui Wang

**Affiliations:** ^1^Key Laboratory for Organic Electronics and Information Displays & Jiangsu Key Laboratory for Biosensors, Institute of Advanced Materials (IAM), Jiangsu National Synergetic Innovation Centre for Advanced Materials (SICAM), Nanjing University of Posts and Telecommunications, Nanjing 210023, China; ^2^Department of Oral Implantology, Nanjing Stomatological Hospital, School of Medicine, Nanjing University, Nanjing 210023, China; ^3^School of Geography and Biological Information, Nanjing University of Posts and Telecommunications, Nanjing 210023, China; ^4^Department of Medical Imaging, Jinling Hospital, School of Medicine, Nanjing University, Nanjing 210002, China

## Abstract

The formation of bacterial biofilms closely associates with infectious diseases. Until now, precise diagnosis and effective treatment of bacterial biofilm infections are still in great need. Herein, a novel multifunctional theranostic nanoplatform based on MnO_2_ nanosheets (MnO_2_ NSs) has been designed to achieve pH-responsive dual-mode imaging and hypoxia-relief-enhanced antimicrobial photodynamic therapy (aPDT) of bacterial biofilm infections. In this study, MnO_2_ NSs were modified with bovine serum albumin (BSA) and polyethylene glycol (PEG) and then loaded with chlorin e6 (Ce6) as photosensitizer to form MnO_2_-BSA/PEG-Ce6 nanosheets (MBP-Ce6 NSs). After being delivered into the bacterial biofilm-infected tissues, the MBP-Ce6 NSs could be decomposed in acidic biofilm microenvironment and release Ce6 with Mn^2+^, which subsequently activate both fluorescence (FL) and magnetic resonance (MR) signals for effective dual-mode FL/MR imaging of bacterial biofilm infections. Meanwhile, MnO_2_ could catalyze the decomposing of H_2_O_2_ in biofilm-infected tissues into O_2_ and relieve the hypoxic condition of biofilm, which significantly enhances the efficacy of aPDT. An *in vitro* study showed that MBP-Ce6 NSs could significantly reduce the number of methicillin-resistant S*taphylococcus aureus* (MRSA) in biofilms after 635 nm laser irradiation. Guided by FL/MR imaging, MRSA biofilm-infected mice can be efficiently treated by MBP-Ce6 NSs-based aPDT. Overall, MBP-Ce6 NSs not only possess biofilm microenvironment-responsive dual-mode FL/MR imaging ability but also have significantly enhanced aPDT efficacy by relieving the hypoxia habitat of biofilm, which provides a promising theranostic nanoplatform for bacterial biofilm infections.

## 1. Introduction

Bacterial infection is a prominent threat for human health. Numerous bacterial infections, including dental caries, cystic fibrosis, pneumonia, otitis media, and especially chronic wounds, usually relate with the formation of bacterial biofilms [1–5]. Biofilm is the aggregated bacterial populations which are usually encapsulated in the extracellular polymeric substances (EPS) and attach to the living or inert surfaces [6, 7]. The compact EPS of bacterial biofilm not only protect themselves from the attack of host immune system but also cause serious antibiotic resistance, which bring great challenge to eradicate bacterial biofilm infections [8, 9]. Specific and sensitive diagnosis of bacterial biofilm infections is essential to effectively treat these diseases [10–12]. Traditional diagnostic methods, such as culture method, biochemistry identification, and polymerase chain reaction (PCR), have been widely used in bacterial biofilm infections diagnosis.However, these *ex vivo* methods generally need invasive tissue ablation, time-consuming procedures, and have low sensitivity [13–15]. Noninvasive and effective detection methods of bacterial biofilm infections *in vivo* are highly desired.

Recently, various imaging technologies have been developed for the detection of bacterial infections. Considered as a sensitive method for disease diagnosis, fluorescence imaging (FLI) has received great attention [15, 16]. Numerous bacteria-targeting fluorescent imaging probes have been designed for FLI of bacterial infections, such as maltodextrin, vancomycin, and *N*-acetylmuramic acids functionalized dyes [17–19]. Nevertheless, the light scattering and absorption in biological tissues usually cause poor penetration of FLI [20, 21]. In comparison, magnetic resonance imaging (MRI) has the advantages of deep tissue penetration and high spatial resolution [22]. Previous work has reported the use of MRI technique to monitor the bacterial infections [23]. However, relatively low sensitivity of MRI limits its application for bacterial infections [22]. Currently, most imaging probes with bacterial detection ability only work in single mode, which usually limits their use due to the intrinsic shortcomings of each imaging mode. Moreover, these imaging probes mentioned above were designed to target planktonic bacteria rather than bacterial biofilms. The dense EPS matrix of biofilm may prevent the penetration of imaging agents and limit their application for bacterial biofilm infections. In addition, currently used imaging probes for bacterial infections usually work in “always-on” mode that may cause significant background signal and reduce their sensitivity. Therefore, FL/MR dual-mode imaging probes with bacterial biofilm responsive and activable ability are very promising.

Due to the encapsulation of EPS and fermentation of bacteria inside biofilm, the biofilm microenvironment has several characteristics, such as the lack of oxygen, low pH, and high level of H_2_O_2_ compared with healthy tissues [24–29]. Hence, biofilm microenvironment is an ideal target for specific diagnosis and treatment of biofilm-related infection diseases [29–31]. Previous studies have demonstrated the use of charge-switchable nanozymes to respond the acidic biofilm microenvironment through bioorthogonal reaction to activate profluorophores and realize specific fluorescence detection of biofilm *in vitro* [24]. Moreover, pH-responsive Au nanoparticles and micellar nanocarriers have also been designed to combat bacterial biofilm infections [32, 33]. Although these studies are promising, they usually work in single mode (diagnosis or treatment) and lack the ability of simultaneous diagnosis and therapy. To the best of our knowledge, biofilm microenvironment-responsive nanotheranostics that combine both activable dual-mode FL/MR imaging and treatment abilities to combat biofilm infections are rarely reported.

**Scheme 1:**
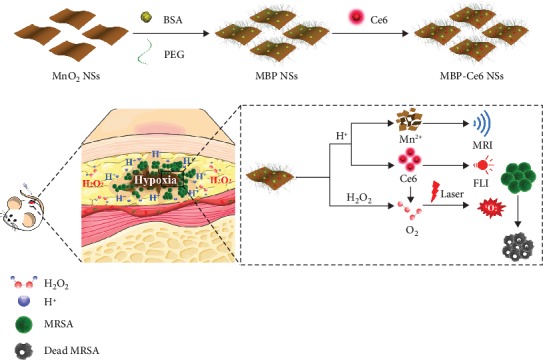
The preparation of MnO_2_-BAS/PEG-Ce6 nanosheets (MBP-Ce6 NSs) and their use for responsive FL/MR imaging and enhanced aPDT of bacterial biofilm infections.

Here, we designed a biofilm microenvironment-responsive theranostic nanoagent (MBP-Ce6 NSs) for dual-mode FL/MR imaging and enhanced aPDT of MRSA biofilm infections. As Scheme 1 shows, MnO_2_ nanosheets (MnO_2_ NSs) were modified with biocompatible BSA and PEG to form MnO_2_-BSA/PEG NSs (MBP NSs). Then, a photosensitizer Ce6 was further loaded to prepare MBP-Ce6 NSs. The fluorescence of Ce6 could be quenched after loading on the surface of MnO_2_ NSs. In acidic biofilm microenvironment, MnO_2_ NSs were decomposed and generate Mn^2+^ ions, which can be used for biofilm responsive T_1_-weighted MRI [21, 33, 34]. Meanwhile, the decomposition of MnO_2_ will also result in the release of Ce6 from the MBP-Ce6 NSs, restoration of the fluorescence of Ce6, and realization of the biofilm-activable fluorescence imaging. Moreover, the released Ce6 can also serve as the photosensitizer for aPDT and generate singlet oxygen (^1^O_2_) under light irradiation. ^1^O_2_ can effectively damage the vital biomacromolecules (DNA, protein, etc.) and has been used for the treatment of cancer and bacterial infection [28, 34, 35]. Since it is difficult for bacteria to develop resistance to ^1^O_2_, aPDT is regarded as an effective approach for the drug-resistant bacterial infection [32, 36]. However, the therapeutic efficacy of aPDT would be restricted by hypoxia condition in biofilm-infected tissues [4, 26]. Due to the high level H_2_O_2_ in biofilm-infected tissues, MnO_2_ NSs can convert the H_2_O_2_ into O_2_ and enhance O_2_-dependent aPDT. MBP-Ce6 NSs were successfully used for acid-activated dual-mode imaging and hypoxia relieving aPDT of MRSA biofilm-infected mice.

## 2. Results and Discussion

### 2.1. Material Preparation and Characterization

**Figure 1:**
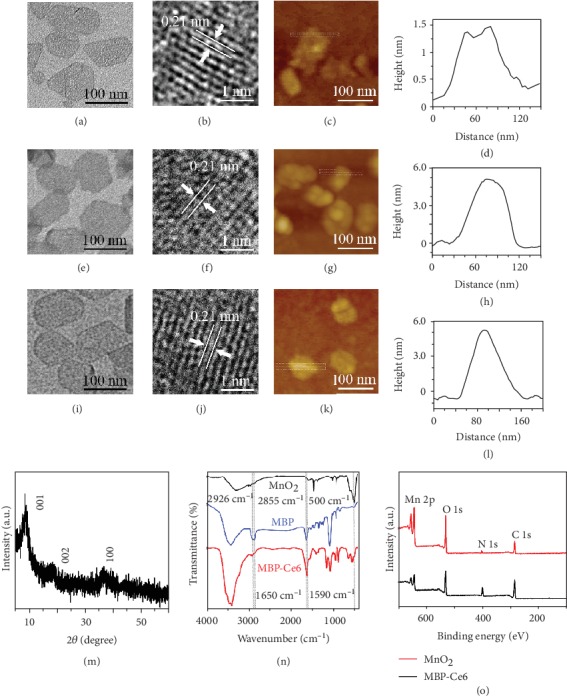
Characterization of MnO_2_ NSs, MBP NSs, and MBP-Ce6 NSs. (a) TEM image, (b) HRTEM image, (c) AFM image, and (d) height profile of MnO_2_ NSs. (e) TEM image, (f) HRTEM image, (g) AFM image, and (h) height profile of MBP NSs. (i) TEM image, (j) HRTEM image, (k) AFM image, and (l) height profile of MBP-Ce6 NSs. (m) XRD pattern of MnO_2_ NSs. (n) FT-IR spectra of MnO_2_ NSs, MBP NSs, and MBP-Ce6 NSs. (o) XPS spectra of MnO_2_ NSs and MBP-Ce6 NSs.

MnO_2_ NSs were synthesized according to the reported method [37]. Manganese (II) chloride reacted with H_2_O_2_ and tetramethylammonium hydroxide (TMA·OH) to form aggregated MnO_2_ NSs (Figure S1, Supporting Information). MnO_2_ NSs were prepared by ultrasonication-assisted exfoliation of the as-prepared MnO_2_ NSs and followed by gradient centrifugation. Transmission electron microscopy (TEM) images (Figure 1(a); Figures S2(a) and (d), Supporting Information) show that the as-prepared MnO_2_ NSs have uniform sheet-like morphology and small average size (<100 nm). As the selected area electron diffraction (SAED) pattern (Figure S3(a), Supporting Information) shows, MnO_2_ NSs have a polycrystalline structure. A high-resolution TEM (HRTEM) image (Figure 1(b)) shows the distinct crystal lattice spacing about 0.21 nm, which can be ascribed to the (202) plane of MnO_2_ [20, 37]. A high-angle annular dark-field scanning transmission electron microscopy (HAADF-STEM) image and energy dispersive spectroscopy (EDS) elemental mapping images (Figures S3(b), (c), (d), and (e), Supporting Information) show homogeneous element distribution in MnO_2_ NSs. Atomic force microscopy (AFM) images (Figure 1(c); Figures S4(a) and (d), Supporting Information) reveal that the average thickness of MnO_2_ NSs is about 1.08 nm, which proves the single-layer structure of MnO_2_ NSs [37]. To enhance the stability and biocompatibility of MnO_2_ NSs, BSA and PEG_3000_-NH_2_ were used to modify the surface of MnO_2_ NSs through the van der Waals interaction and Mn-N coordinate bonding to form MBP NSs [20, 38]. Next, Ce6 was further loaded on the surface of MBP NSs by similar noncovalent interactions [39]. As the TEM images (Figure 1(e) and (i); Figures S2(b), (c), (e), and (f), Supporting Information) and HRTEM images (Figure 1(f) and (j)) show, the morphology, size, and crystal structure of the MBP NSs and MBP-Ce6 NSs have no obvious change compared with MnO_2_ NSs. The thickness of MBP NSs increased to about 5.07 nm (Figure 1(g) and (h); Figures S4(b) and (e), Supporting Information) due to the existence of BSA and PEG. After loading with Ce6, the thickness of MBP-Ce6 NSs further increased to about 5.36 nm (Figure 1(k) and (l); Figures S4(c) and (f), Supporting Information).

The crystal structure of MnO_2_ NSs was determined by X-ray diffraction (XRD). Based on the XRD pattern of MnO_2_ NSs shown in Figure 1(m), three diffraction peaks, located at 8.7°, 18.8°, and 37.0°, can be assigned to the (001), (002), and (100) planes, respectively [37]. The Fourier transform infrared spectroscopy (FT-IR) and X-ray photoelectron spectroscopy (XPS) were also carried out to further investigate the compositions of MBP-Ce6 NSs. As shown in Figure 1(n), the IR absorption bands of MnO_2_ NSs around 500 cm^−1^ belong to the Mn-O stretching vibrations [37]. The IR absorption bands of MBP NSs at 2926 cm^−1^ and 2855 cm^−1^ can be ascribed to the C-H stretching vibrations from the PEG [40]. The IR adsorption bands of MBP-Ce6 NSs near 1650 cm^−1^ and 1590 cm^−1^ can be assigned to the C=O bending vibrations from BSA and the N-H bending vibrations from Ce6, respectively [40, 41]. The XPS survey spectrum (Figure 1(o)) of MnO_2_ NSs shows the binding energy peaks located at 654 eV and 642 eV, which belong to Mn (IV) 2p_1/2_ and Mn (IV) 2p_3/2_ of MnO_2_ [42]. For the MBP-Ce6 NSs, the intensity of Mn 2p peaks decreases, while the intensity of N 1s (400 eV) and C 1s (285 eV) peaks increases, indicating the presence of BSA, PEG, and Ce6 on the surface of MnO_2_ NSs. As Figure S5(a) (Supporting Information) shows, the hydrodynamic diameter of MBP-Ce6 NSs is larger than that of MnO_2_ and MBP NSs, suggesting the successful surface modification. The zeta potential of MnO_2_ NSs is about -7.62 mV (Figure S5b, Supporting Information) [37]. After surface modification with BSA and PEG, it increased to -2.05 mV because of the existence of electroneutral PEG. While further loaded with Ce6, the zeta potential of MBP-Ce6 NSs decreased to about -35.70 mV owing to the negative charge of Ce6 [41].

**Figure 2:**
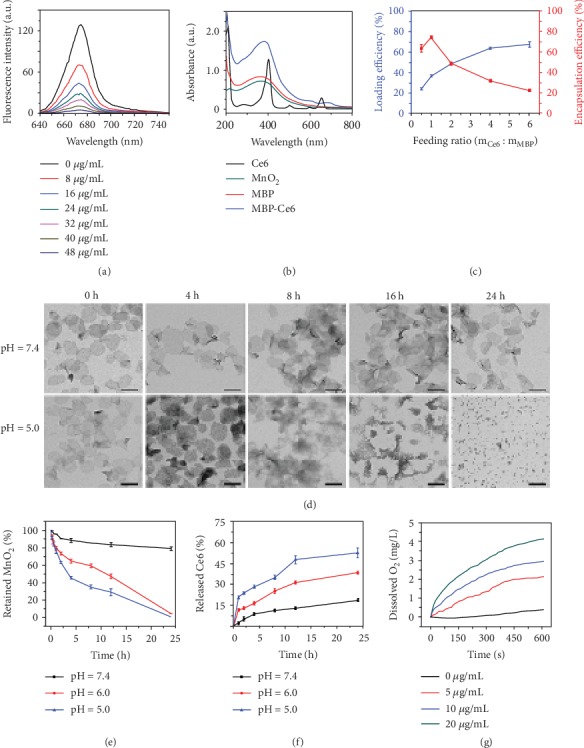
Characterization and drug release of MBP-Ce6 NSs. (a) The fluorescence spectra of Ce6 aqueous solutions (20 *μ*g/mL) after being mixed with MnO_2_ NSs (0, 6, 12, 18, 24, 30, 36, and 48 *μ*g/mL). (b) The UV-Vis-NIR spectra of Ce6, MnO_2_ NSs, MBP NSs, and MBP-Ce6 NSs. (c) The loading efficiency and encapsulation efficiency of Ce6 at different feeding ratios of Ce6 and MnO_2_ NSs. (d) The TEM images of MBP-Ce6 NSs after being stored in PBS at different conditions. The scale bar is 100 nm for all TEM images. (e) The time-dependent degradation of MBP NSs (40 *μ*g/mL) in PBS at different pH values. (f) Released Ce6 from MBP-Ce6 NSs over time at different conditions. (g) The time-dependent concentration change of dissolved O_2_ in H_2_O_2_ solutions (50 *μ*M) after being mixed with MBP-Ce6 NSs at different concentrations (0, 5, 10, and 20 *μ*g/mL). Error bars indicate s.d. (*n* = 3).

Ce6 loading and fluorescence quenching abilities of MBP NSs were also investigated. As shown in Figure 2(a), the fluorescence intensity of Ce6 (20 *μ*g/mL) nearly diminished after being mixed with MBP NSs (MnO_2_: 48 *μ*g/mL) due to the quenching of MnO_2_ NS [43, 44]. The ultraviolet-visible-near infrared (UV-Vis-NIR) absorption spectrum (Figure 2(b)) of MBP-Ce6 NSs shows that the absorbance of the characteristic peaks for Ce6 near 400 and 650 nm is much higher than that of MBP NSs and MnO_2_ NSs, suggesting the successful loading of Ce6 on MBP NSs [45]. As illustrated in Figure 2(c), the loading efficiency of Ce6 reaches as high as 67.5% at the feeding ratio of 6 : 1 (m_Ce6_ : m_MBP_), while the corresponding encapsulation efficiency is about 22.5%, indicating good Ce6-loading capacity of MBP NSs.

Since MnO_2_ nanostructures could be decomposed in acidic aqueous solutions and release Mn^2+^, MnO_2_ NSs have been used as pH-responsive drug delivery platforms [46]. The pH-responsive release of Ce6 by MBP-Ce6 NSs was then studied. The morphology change of MBP-Ce6 NSs after being incubated in phosphate-buffered saline (PBS) with different pH values was studied by using TEM. As shown in Figure 2(d), the MBP-Ce6 NSs gradually broke up in acidic condition (pH = 5.0) after 4 h incubation, while those in neutral PBS (pH = 7.4) still kept intact morphology even after 24 h storage. The decomposition of MBP-Ce6 NSs was also studied by using the UV-Vis-NIR absorption spectroscopy. As indicated in Figure 2(e), the degradation of MBP-Ce6 NSs was less than 20% in PBS at pH = 7.4 after 24 h storage at room temperature. In comparison, nearly all the MBP-Ce6 NSs were decomposed in acidic PBS at pH = 5.0 and 6.0. The acid-induced decomposition of MBP-Ce6 NSs could trigger the fast release of Ce6. The amount of released Ce6 and the fluorescence recovery were studied by the UV-Vis-NIR absorption spectroscopy and fluorescence spectroscopy, respectively. As illustrated in Figure 2(f), the release of Ce6 is much faster in acidic conditions than in the neutral condition. In acidic PBS (pH = 5.0), the released Ce6 reached 52.64% after 24 h incubation, while that in neutral PBS (pH = 7.4) was only 18.69%. Figure S6 (Supporting Information) further indicates that the fluorescence of Ce6 reached the highest intensity after 12 h storage, and the release of the Ce6 in acidic conditions is much faster than that in neutral condition.

Hypoxia is another typical characteristic of the microenvironment in bacterial biofilm-infected tissues [47, 48]. Since oxygen is an essential factor in the process of PDT, the hypoxia condition can significantly limit the therapeutic efficacy of PDT. Fortunately, H_2_O_2_ usually exists at a relatively high level in bacteria-infected tissues and can be used as a potential oxygen source [28, 49–51]. Therefore, the peroxidase-like ability of MBP-Ce6 NSs was investigated. After MBP-Ce6 NSs were dispersed in H_2_O_2_ aqueous solution (50 *μ*M), the concentration of dissolved O_2_ in the mixture changed according to the concentration of MnO_2_ (Figure 2(g)), which indicates that MBP-Ce6 NSs could effectively decompose H_2_O_2_ to release O_2_. The generation of O_2_ could relieve the hypoxia of bacterial biofilm-infected tissues and may further enhance the efficacy of oxygen-dependent aPDT. Therefore, the singlet oxygen (^1^O_2_) production of MBP-Ce6 NSs in hypoxic conditions with the presence of H_2_O_2_ was measured and evaluated by using 2′,7′-dichlorodihydrofluorescein diacetate (DCFH-DA) as the fluorescent reactive oxygen species (ROS) indicator [52–54]. As shown in Figure S7(a) (Supporting Information), the ^1^O_2_ generation efficiency of MBP-Ce6 NSs is less effective than free Ce6 in normoxic condition due to the fluorescence quenching of MnO_2_ NSs. In contrast, MBP-Ce6 NSs can efficiently produce high-level ^1^O_2_ after laser irradiation in hypoxic conditions with the presence of H_2_O_2_ similar to the biofilm microenvironment, while Ce6, H_2_O_2_, Ce6 + H_2_O_2_, and MBP-Ce6 NSs generate neglectable ^1^O_2_ (Figure S7(b), Supporting Information), suggesting that MBP-Ce6 NSs can serve as efficient agent during aPDT process in hypoxic conditions. These results mentioned above are consistent with the elevated concentration of O_2_ shown in Figure 2(g).

The colloidal stability and cytotoxicity of MBP-Ce6 NSs were assessed before further biological applications. After being dispersed in various conditions (H_2_O, PBS, and Dulbecco's modified Eagle's medium (DMEM)), MBP-Ce6 NSs showed no obvious aggregation (Figure S8(a), Supporting Information). Moreover, the change of their hydrodynamic diameters within 24 h was neglectable (Figure S8(b), Supporting Information), indicating excellent colloidal stability of MBP-Ce6 NSs. Then, MPB-Ce6 NSs with different concentrations (MnO_2_: 0, 20, 40, 80, 160, and 320 *μ*g/mL; Ce6: 0, 16, 32, 64, 128, and 256 *μ*g/mL) were cultivated with human prostatic stromal myofibroblast (WPMY-1) cells for 24 h. Results show that MBP-Ce6 NSs have little cytotoxicity even at high concentration (Figure S9, Supporting Information).

### 2.2. In Vitro Photodynamic Treatment of MRSA Biofilms

**Figure 3:**
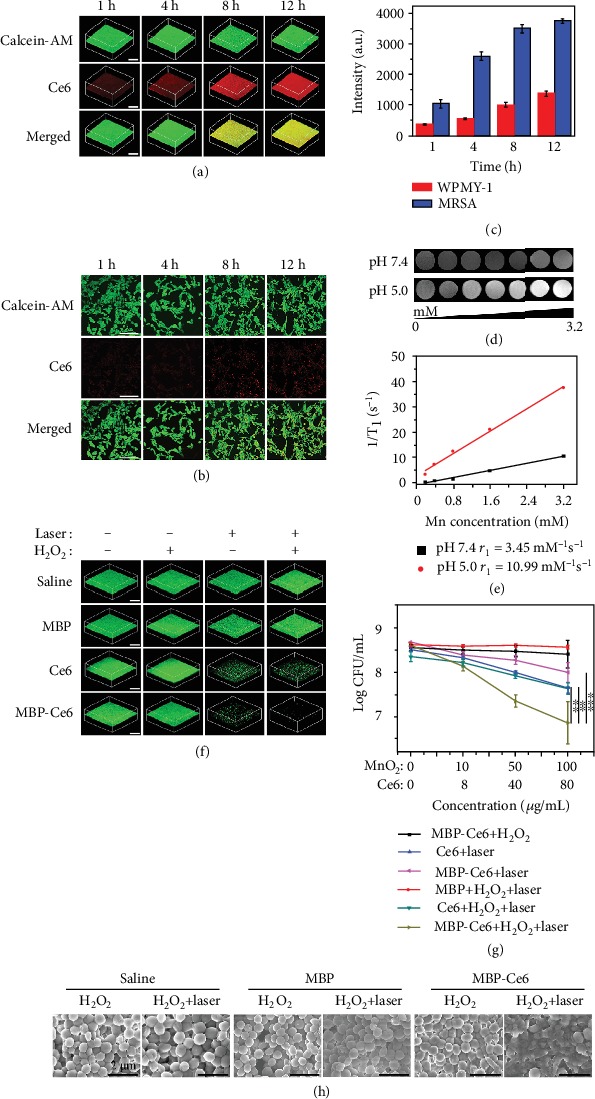
*In vitro* Ce6 releasing behavior of MBP-Ce6 NSs and photodynamic treatment of MRSA biofilms. (a) 3D CLSM images of MRSA biofilms after being incubated with MBP-Ce6 (MnO_2_ NSs: 100 *μ*g/mL; Ce6: 80 *μ*g/mL) at different times. The scale bar is 200 *μ*m. (b) CLSM images of WPMY-1 cells after being treated with MBP-Ce6 (MnO_2_ NSs: 100 *μ*g/mL; Ce6: 80 *μ*g/mL) at different times. The green fluorescence and the red fluorescence originate from Calcein-AM and Ce6, respectively. The scale bar is 200 *μ*m. (c) The fluorescence intensity of Ce6 after MBP-Ce6 NSs (MnO_2_ NSs: 100 *μ*g/mL; Ce6: 80 *μ*g/mL) incubated with MRSA biofilms and WPMY-1 cells at different times. (d) T_1_-weighted MR images of MBP-Ce6 NSs solutions with different concentrations and pH values in PBS. (e) The transverse relativities (*r*_1_) for MBP-Ce6 NSs under different pH values in PBS. (f) 3D CLSM images of live bacteria (green fluorescence) inside MRSA biofilms after various treatments and stained with Calcein-AM. The scale bar is 200 *μ*m. (g) The CFU number of bacteria inside MRSA biofilms after different treatments. ^∗∗^*p* < 0.01 and ^∗∗∗^*p* < 0.001 (two-tailed Student's *t*-test). (h) SEM images of MRSA biofilms treated with saline, MBP NSs (MnO_2_ NSs: 100 *μ*g/mL), and MBP-Ce6 NSs (MnO_2_ NSs: 100 *μ*g/mL; Ce6: 80 *μ*g/mL) under different conditions. The scale bar is 2 *μ*m. The concentration of H_2_O_2_ is 50 *μ*M. The laser irradiation was carried out by using the power density of 20 mW/cm^2^ at 635 nm for 30 min. Error bars indicate s.d. (*n* = 3).

To investigate the Ce6 release and fluorescence imaging abilities of MBP-Ce6 NSs in bacterial biofilms *in vitro*, MRSA biofilms were used as a model. For comparison, WPMY-1 cells were adopted as the control to mimic the normal environment condition. After being treated with MBP-Ce6 NSs (MnO_2_ NSs: 100 *μ*g/mL; Ce6: 80 *μ*g/mL) at different times, gradually increased red fluorescence (Ce6) in MRSA biofilms could be observed from the 3D confocal laser scanning microscopy (3D CLSM) images during incubation (Figure 3(a)), while no distinct fluorescence of Ce6 could be seen for WPMY-1 cells at the same experimental conditions (Figure 3(b)). The fluorescence intensity of Ce6 incubated with MRSA biofilms is about 2.5 times higher than that of WPMY-1 cells at 12 h (Figure 3(c)), suggesting MBP-Ce6 NSs have specific biofilm microenvironment-responsive releasing ability. The size decrease of MBP-Ce6 NSs may improve the penetration of Ce6 in biofilms and further enhance their PDT effect.

The MR imaging ability of MBP-Ce6 NSs in bacterial biofilm microenvironment was further evaluated. As shown in Figure 3(d), the T_1_-weighted MR signal of MBP-Ce6 NSs gradually increased in acidic solution (pH = 5.0) with the increase of their concentration, while that in neutral condition (pH = 7.4) showed a much weaker MR signal. Figure 3(e) indicates that the transverse relativity (*r*_1_) of MBP-Ce6 NSs after being incubated in acidic buffer (pH = 5.0) for 24 h is 10.99 mM^−1^ s^−1^, which is about 3 times of that in neutral buffer (3.47 mM^−1^ s^−1^, pH = 7.4). Such significantly enhanced MR signal proves that MBP-Ce6 NSs could serve as pH-responsive MRI contrast agents.

Then, the efficiency of MBP-Ce6 NSs for the treatment of MRSA biofilms *in vitro* was evaluated. Based on the 3D CLSM images shown in Figure 3(f), MRSA biofilms treated by MBP-Ce6 NSs with both laser irradiation and H_2_O_2_ have the lowest green fluorescence, indicating their excellent therapeutic effect. In contrast, MRSA biofilms in other groups with different treatments (Ce6 + laser, H_2_O_2_ + laser, and MBP-Ce6 NSs + laser) showed much stronger fluorescence, suggesting limited antibiofilm effect. The *in vitro* aPDT efficiency of MBP-Ce6 NSs for MRSA biofilms was also studied. As shown in Figure 3(g), the colony-forming units (CFU) number of MRSA biofilms treated by MBP-Ce6 NSs (MnO_2_ NSs: 100 *μ*g/mL, Ce6: 80 *μ*g/mL) and H_2_O_2_ (50 *μ*M) with the laser irradiation decreased by 1.5 log (97%). In comparison, the bacteria inactivation efficiency is less than 0.6 log (75%) for other groups (Ce6 + laser, Ce6 + H_2_O_2_ + laser, and MBP-Ce6 + laser). For the MBP-Ce6 + H_2_O_2_ and MBP + H_2_O_2_ + laser groups, no obvious antibacterial effect can be observed. These results prove that the MBP-Ce6 NSs can achieve excellent antibiofilm performance by aPDT with the presence of H_2_O_2_, which may be converted into O_2_ by the MnO_2_ catalyzing and facilitate the generation of ^1^O_2_. To verify this assumption, the generation of ^1^O_2_ in MRSA biofilms after different treatments was tested. As indicated in Figure S10 (Supporting Information), MRSA biofilms treated by MBP-Ce6 NSs with laser and H_2_O_2_ showed much stronger fluorescence than those treated by saline, Ce6, and MBP with laser and H_2_O_2_, suggesting a higher level of ^1^O_2_. SEM images (Figure 3(h)) reveal that the MRSA biofilms in MBP-Ce6 + laser + H_2_O_2_ group show distinctly structure damage, which can also be observed from the images of MRSA biofilms stained by crystal violet with decrease of relative biofilm biomass (Figure S11, Supporting Information).

### 2.3. Microenvironment-Responsive Dual-Mode Imaging of MRSA Biofilm Infection

**Figure 4:**
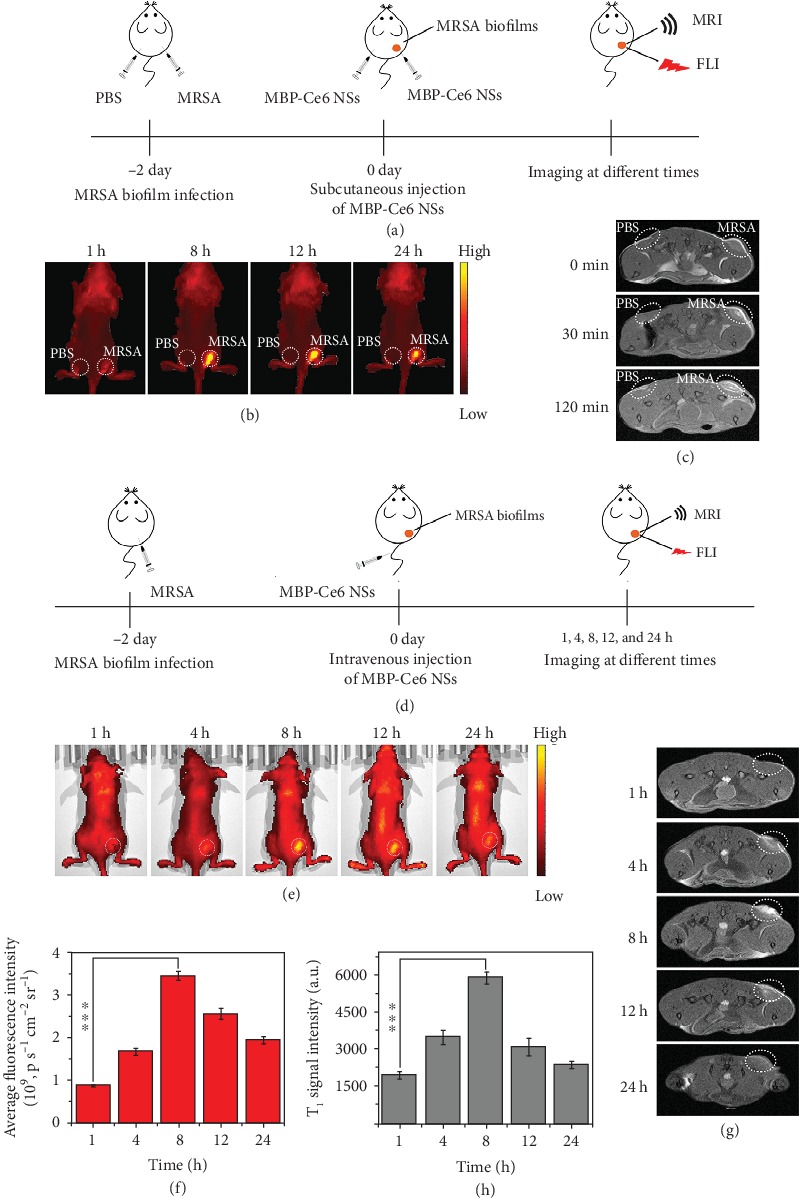
*In vivo* fluorescence imaging (FLI) and magnetic resonance imaging (MRI) of MRSA biofilm infections by using MBP-Ce6 NSs. (a) Schematic illustration for the detection of MRSA biofilms by *in situ* injection of MBP-Ce6 NSs (50 *μ*L; MnO_2_: 50 *μ*g/mL; and Ce6: 40 *μ*g/mL) both in the left thigh (normal tissue) and the right thigh (infected tissue). (b) Fluorescence images and (c) T_1_-weighted MR images of the infected mice at different times. (d) Schematic illustration of the process for the detection of MRSA biofilms by i.v. injection of MBP-Ce6 NSs. (e) Fluorescence images and the (f) fluorescence intensity of MRSA-infected mice at different times postinjection. (g) T_1_-weighted MR images shown in cross-section and (h) the intensity of the T_1_-weighted MR signal of the MRSA biofilm-infected tissues at different times postinjection. White dashed circles in (e) and (g) denote MRSA biofilm-infected tissues. ^∗∗∗^*p* < 0.001 (two-tailed Student's *t*-test). All data are presented as means ± s.d. (*n* = 3).

In order to evaluate the *in vivo* imaging capability of MBP-Ce6 NSs, MRSA biofilm-infected mice models were constructed by subcutaneously injection of MRSA dispersions into the right thigh (biofilm infected) of each mouse (Figure 4(a)). In comparison, PBS dispersion was also used to treat the left thigh (control) of the same mice. Two days later, MRSA biofilm infections with significant abscesses (Figure S12(a), Supporting Information) appeared and could be confirmed by wound blotting (Figure S12(b), Supporting Information) [55, 56]. The *in vivo* imaging of MRSA biofilms was first studied by *in situ* injection of MBP-Ce6 NSs.

As illustrated in Figure 4(b) and (c), and Figure S13 (Supporting Information), the biofilm-infected tissues (right thigh) showed significantly enhanced fluorescence and T_1_-weighted MR signals after local injection of MBP-Ce6 NSs compared with normal tissues (left thigh), which suggests that detection of the MRSA biofilm infections could be achieved by MBP-Ce6 NSs. To further examine the *in vivo* FL/MR dual-mode imaging ability, MBP-Ce6 NSs were used to detect MRSA biofilm infections by intravenous (i.v.) injection (Figure 4(d)). Figure 4(e) and (f) show that the fluorescence of the infected tissues in mice treated by i.v. injection of MBP-Ce6 NSs increased gradually and peaked at the 8th hour postinjection. After 24 h postinjection, the fluorescence signal in biofilm-infected tissues was much stronger than other organs (Figure S14, Supporting Information), indicating specific accumulation in infected tissues due to the EPR effect and pH-responsive decomposition of MBP-Ce6 NSs [29, 42]. Meanwhile, the T_1_-weighted MR signal of the MRSA biofilm infections also increased obviously during the first 8 h and gradually decreased afterwards (Figure 4(g) and (h)), similar to the change of fluorescence signal. As Figure S15 (Supporting Information) shows, the T_1_-weighted MR signal in the kidneys was much stronger than that in other organs during 24 h postinjection and gradually became weaker after the 8th hour postinjection, suggesting that the Mn^2+^ ions released from MBP-Ce6 NSs could be quickly excreted through the kidney [45]. In addition, we also found that the amount of Mn in biofilm-infected tissues is much higher than that in the other organs, indicating the specific accumulation of MBP-Ce6 NSs in MRSA biofilm-infected tissues (Figure S16, Supporting Information). Furthermore, the content of Mn in the organs quickly decreased to low level within 48 h, which demonstrates that MBP-Ce6 NSs could be quickly excreted from the body after decomposition within relatively short periods [42].

### 2.4. Relieving Hypoxia for Enhanced PDT of MRSA Biofilm Infection

During the development of bacterial biofilms, O_2_ is consumed by both the bacteria inside the biofilms and the surrounding inflammatory cells, while the diffusion of O_2_ into the biofilms is hampered by the existence of the dense EPS, which together cause the hypoxia in bacterial biofilm microenvironment [47, 57]. It is known that PDT is an oxygen-dependent process and the therapeutic efficacy can be greatly restricted by the hypoxic conditions. Hence, the hypoxia microenvironment in biofilms is an intrinsic barrier for aPDT treatment. As reported, excess H_2_O_2_ usually exists in biofilm-infected tissues [4, 28], in principle, which could be converted to O_2_ with the catalysis of MnO_2_. Herein, the potential of MBP-Ce6 NSs to generate O_2_ and relieve the hypoxia of biofilm was investigated in a mice model. After i.v. injection with PBS, Ce6, MBP NSs, and MBP-Ce6 NSs, the biofilm-infected tissues of mice were harvested at 8th hour posttreatment and stained with fluorescent dye-labeled HIF-1*α* antibody [58].

**Figure 5:**
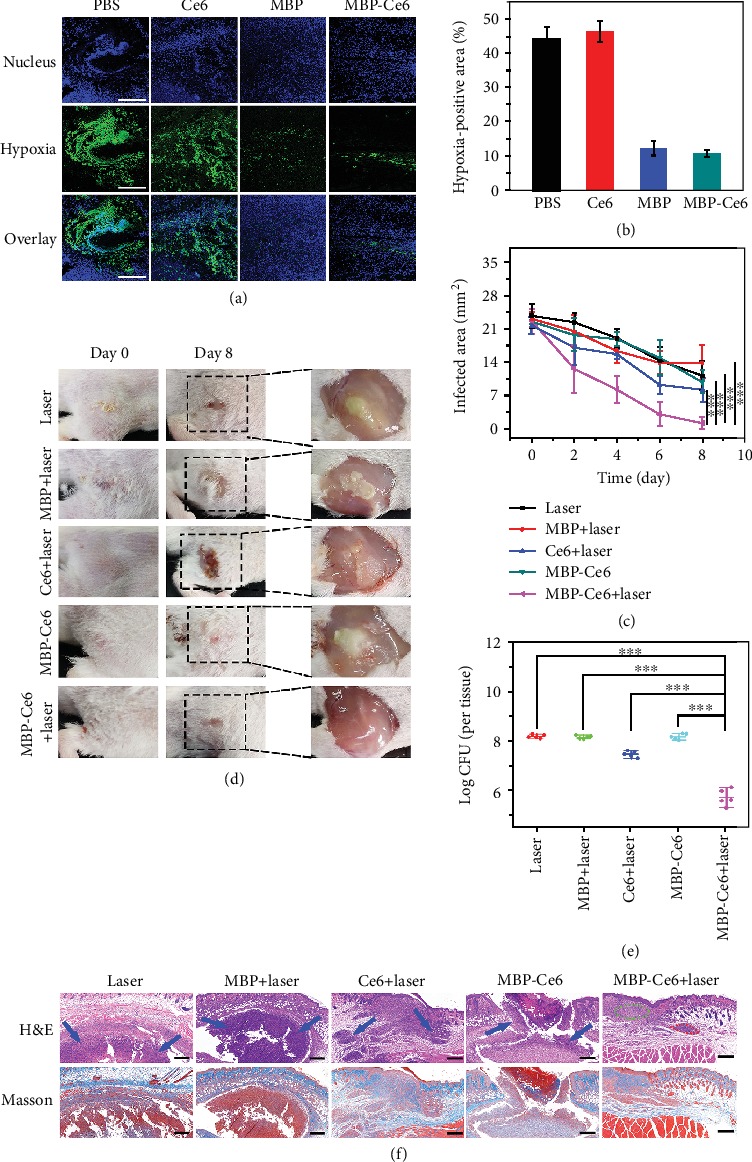
Photodynamic treatment of MRSA biofilm infections *in vivo*. (a) CLSM immunofluorescence images of the infected tissue slices from the mice after i.v. injection with PBS, Ce6, MBP NSs, and MBP-Ce6 NSs for 8 h, respectively. The hypoxic condition was indicated by staining the slices with HIF-1*α* antibody (green) and 4′,6-diamidino-2-phenylindole (DAPI, blue). The scale bar is 200 *μ*m. (b) Semiquantification of the hypoxia-positive area in biofilm-infected tissues after various treatments. (c) Biofilm-infected tissue areas of mice after different treatments at different times. (d) Photographs of the biofilm-infected tissues from the mice with various treatments at different times. (e) CFU of MRSA in biofilm-infected tissues at 8th day posttreatment. (f) Histological analysis of the biofilm-infected tissues by H&E and Masson's trichrome staining. Blue arrows denote the inflammatory cell infiltration. Green circle indicates the proliferation of fibroblast and neovascularization. Red circle indicates granulomatous inflammation. The scale bar is 200 *μ*m. ^∗∗∗^*p* < 0.001 (two-tailed Student's *t*-test). All data are presented as means ± s.d. (*n* = 5).

As shown in Figure 5(a), CLSM images indicate that the MRSA biofilm-infected tissues treated with PBS or Ce6 showed a larger hypoxia-positive area (green), while those treated with MBP NSs or MBP-Ce6 NSs exhibited a limited hypoxia-positive area. The semiquantification analysis results (Figure 5(b)) further confirm that the treatment by MBP NSs or MBP-Ce6 NSs could effectively relieve the hypoxia conditions inside MRSA biofilm-infected tissues. The *in vivo* therapeutic efficacy of aPDT by using MBP-Ce6 NSs was further studied. The mice with MRSA biofilm infections were divided into different groups according to different treatments: laser irradiation (group 1, laser), MBP NSs with laser irradiation (group 2, MBP + L), Ce6 with laser irradiation (group 3, Ce6 + L), MBP-Ce6 NSs (group 4, MBP-Ce6), and MBP-Ce6 NSs with laser irradiation (group 5, MBP-Ce6 + L). After i.v. injection of the agents (dose of MnO_2_ = 5 mg/kg; dose of Ce6 = 4 mg/kg) for 8 h, laser irradiation under 635 nm laser (20 mW/cm^2^) was performed in groups 1, 2, 3, and 5 for 30 min. At therapeutic day 8, the mice in group 5 (MBP-Ce6 NSs + laser) showed significantly reduction of the infected area compared with the other four groups (Figure 5(c)). The abscesses and wound beds of the mice in group 5 disappeared, while the mice with other treatments (groups 1, 2, 3, and 4) still had a large area of infection (Figure 5(d)). In addition, the infected tissues were harvested and the number of viable bacteria inside biofilm-infected tissues was evaluated at 8th day posttreatment. Figure 5(e) shows that the bacterial inactivation efficiency of group 5 (MBP-Ce6 NSs + laser) is about 2.5 log (99.7%), much higher than those in group 1 (0%), group 2 (0%), group 3 (80.1%), and group 4 (0%). As shown in the hematoxylin-eosin (H&E) staining images (Figure 5(f); Figure S17, Supporting Information), severe inflammation with massive inflammatory cells infiltration still existed in infected tissues of the mice in groups 1-4 (indicated by blue arrows). In contrast, the infected tissues in group 5 (MBP-Ce6 NSs + laser) showed obvious proliferation of fibroblast cells, neovascularization (indicated by green cycles), and a little granulomatous inflammation (indicated by red cycles). Masson's trichrome staining images further indicate the formation of intact subcutaneous tissues and the appearance of more collagen fibers (blue) in group 5 than the other groups (groups 1, 2, 3, and 4), suggesting better recovery of the infected tissues. These results demonstrated that MBP-Ce6 NSs have excellent aPDT efficacy for MRSA biofilm infections, which should be ascribed to the hypoxia-relief in the biofilm microenvironment.

Furthermore, long-term toxicity of MBP-Ce6 NSs to mice was also studied. As shown in Figure S18(a) (Supporting Information), H&E staining images of major organs from the mice after i.v. injection of MBP-Ce6 NSs show no noticeable damage or inflammatory lesion. Figure S18(b) (Supporting Information) indicates that the body weights of the mice treated by MBP-Ce6 NSs steadily increased and had no obvious difference from the mice treated by PBS. The hematology assay results (Figures S19 (c-j), Supporting Information) further show that all serum biochemical parameters and blood routine examination parameters of the mice treated by MBP-Ce6 NSs are similar with the control group at 28 d postinjection, further indicating low toxicity of MBP-Ce6 NSs at the dose used in this study [59].

## 3. Conclusion

In summary, we designed multifunctional nanotheranostics (MBP-Ce6 NSs) for biofilm microenvironment-responsive imaging and treating of MRSA biofilm infections. MBP-Ce6 NSs could be decomposed in the acidic microenvironment in biofilm-infected tissues and subsequently release Ce6 and Mn^2+^ ions. The FL signal from Ce6 and MR signal from Mn^2+^ could be simultaneously activated during the decomposition of MBP-Ce6 NSs. Experimental results show that MBP-Ce6 NSs can achieve FL/MR dual-mode imaging for specific and sensitive detection of MRSA biofilm infections. On the other hand, the hypoxic condition in biofilm microenvironment could be relieved by MnO_2_-triggered decomposition of endogenous H_2_O_2_ in biofilm-infected tissues, while hypoxia-associated resistance of biofilm toward aPDT could be overcome. After being treated by MBP-Ce6 NSs with laser irradiation, the number of viable MRSA from biofilm-infected tissues in mice could be reduced by 2.5 log (99.7%), which is much lower than the control groups, and the recovery of the infected tissues could be promoted. Furthermore, the MBP-Ce6 NSs showed no appreciable toxicity in the *in vivo* study, indicating their potential use in biomedicine. Therefore, this study provides a promising theranostic nanoplatform with potential use for specific detection and treatment of bacterial biofilm infections.

## 4. Materials and Methods

### 4.1. Materials

Manganese chloride tetrahydrate (MnCl_2_•4H_2_O), tetramethylammonium hydroxide solution (TMA, 1.0 M), bovine serum protein (BSA), and 2′,7′-dichloroflourescin diacetate (DCFH-DA) were purchased from Sigma-Aldrich. NH_2_-PEG_3000_ and chlorin e6 (Ce6) were purchased from JenKem Technology and Frontier, respectively. The hydrogen peroxide (H_2_O_2_, 30 wt.%) solution was purchased from Sinopharm Chemical Reagent Co., Ltd. Dulbecco's modified Eagle's medium (DMEM) and fetal bovine serum (FBS) were bought from KeyGen BioTech and Gibco, respectively. HIF-1*α* antibody (mouse monoclonal antibody) and FITC-labeled goat anti-mouse IgG were purchased from Beyotime Biotechnology. All solutions were prepared by using ultrapure water (18.2 M*Ω*).

### 4.2. Characterization

TEM images were obtained by using an HT7700 transmission electron microscope (Hitachi, Japan). Anatomic force microscope (AFM, Nanoscope IIIa, Bruker, USA) was used to measure the thickness of the nanosheets. X-ray diffraction (XRD) patterns were recorded on a D8 ADVANCE X-ray diffractometer (Bruker, Germany) with Cu K*α* radiation. The ultraviolet-visible-infrared (UV-Vis-NIR) absorption spectra were recorded on a UV-3600 spectrophotometer (Shimadzu, Japan). Fluorescence spectra (FL) were recorded on a Shimadzu RF-5301 PC fluorescence spectrophotometer. The concentration of O_2_ in solutions was measured by using a dissolved oxygen detection probe (HACH, China). Photodynamic therapy (PDT) was performed by using a 635 nm continuous-wave semiconductor laser (BWT Beijing, China). The power density of laser irradiation was measured by using a digital power meter (PM100D, Thorlabs, USA). Dynamic light scattering (DLS) and zeta potential were performed on a ZetaPALS Potential Analyzer (Brookhaven Instruments, USA).

### 4.3. Synthesis of MnO_2_ NSs

MnO_2_ nanosheets (MnO_2_ NSs) were prepared based on previous study. Twelve-milliliter TMA solution (0.1 M) and 2 mL H_2_O_2_ (30 wt.%) were mixed with 6 mL ultrapure water as solution A. MnCl_2_•4H_2_O (0.6 g) was dissolved in 10 mL H_2_O as solution B. Then, solution A was quickly mixed with solution B and the color of the mixture became dark brown indicating that the Mn^2+^ was oxidized to Mn^4+^. After stirring overnight, the aggregated MnO_2_ NSs were washed with ethanol and water for three times, respectively, and were then dispersed in 30 mL ultrapure water. To prepare single-layer MnO_2_ NSs, the as-prepared MnO_2_ aggregations were exfoliated by ultrasonication (Yimaneili, 950 W, 25 kHz) for 10 h in an ice-bath. The mixtures were centrifuged at 12000 rpm for 45 min to separate large aggregations and were further centrifuged at 18000 rpm for 90 min to obtain the single-layer MnO_2_ NSs. This step was repeated for three times. The MnO_2_ NSs were dispersed in ultrapure water and then stored at 4°C. The concentration of MnO_2_ NSs was determined by using an inductively coupled plasma atomic emission spectrometer(ICP-AES, Optima 5300DV, PerkinElmer).

### 4.4. Preparation of MnO_2_-BSA/PEG-Ce6 NSs

MnO_2_ NSs (5 mg) and BSA (100 mg) were dispersed in 15 mL water and stirred for 12 h. Then, the mixture was centrifuged at 16000 rpm for 45 min to remove any large aggregations. The MnO_2_-BSA nanosheets were dispersed in 15 mL water and mixed with 50 mg PEG_3000_-NH_2_ by stirring for 12 h. The mixture was centrifuged at 16000 rpm for 45 min and washed with water for three times to get MnO_2_-BSA/PEG nanosheets (MBP NSs). For Ce6 loading, MBP NSs and Ce6 aqueous dispersions were mixed in an orbital shaker for 12 h and then centrifuged at 16000 rpm for 45 min to obtain MnO_2_-BSA/PEG-Ce6 nanosheets (MBP-Ce6 NSs). MBP-Ce6 NSs aqueous dispersions were stored at 4°C.

The loading efficiency and encapsulation efficiency of Ce6 by MBP NSs were determined according to the following equations

(1) $$\begin{array}{c} \text{Ce}6\text{ loading efficiency}\left(\%\right)={\text{W}}_{1}/{\text{W}}_{2}\times 100\% \end{array}$$

(2) $$\begin{array}{c} \text{Ce}6\text{ encapsulation efficiency}\left(\%\right)=\left({\text{W}}_{\text{t}}-{\text{W}}_{\text{s}}\right)/\left({\text{W}}_{\text{t}}\right)\times 100\% \end{array}$$

where *W*_1_ is the weight of Ce6 loaded in MBP-Ce6 NSs, *W*_2_ is the total weight of MBP-Ce6 NSs, *W*_t_ represents the total weight of Ce6 initially added, and *W*_s_ represents the weight of Ce6 remaining in the supernatant.

### 4.5. Degradation and Drug Release Study of MBP-Ce6 NSs

To study the degradation process, MBP-Ce6 NSs were dispersed in PBS solutions with different pH values (5.0, 6.0, and 7.4). At given time points, the MBP-Ce6 NS PBS dispersions were collected and quantified by using UV-Vis absorption spectra. For Ce6 release studies, the MBP-Ce6 NSs were dispersed in PBS solutions with different conditions (pH 7.4 in PBS, pH 5.0 in PBS, pH 7.4 in PBS with 50 *μ*M H_2_O_2_, and pH 5.0 in PBS with 50 *μ*M H_2_O_2_). The amount of released Ce6 was determined by using UV-Vis absorption spectra, and the fluorescence recovery was monitored by using fluorescence spectra.

### 4.6. Detection of Singlet Oxygen during PDT

2′, 7′-Dichlorofluorescein diacetate (DCFH-DA) was used to detect the generation of singlet oxygen. In order to detect the photodynamic effect of MBP-Ce6 NSs in hypoxia conditions, O_2_ were removed from the PBS solutions by bubbling with N_2_ for 30 min. Then, DCFH-DA (10 mg/mL, 5 *μ*L) and Ce6 (1 mg/mL, 10 *μ*L) or MBP-Ce6 (containing 10 *μ*g Ce6) were added into 3 mL PBS solutions either with or without H_2_O_2_ (50 *μ*M), after being irradiated with a 635 nm laser at the power density of 20 mW/cm^2^. The fluorescence spectra of these solutions were collected at different times.

### 4.7. Cytotoxicity of MnO_2_ NSs and MBP-Ce6 NSs

A LDH-Cytotoxicity Colorimetric Assay Kit (BioVision) was used to evaluate the cytotoxicity of MnO_2_ NSs and MBP-Ce6 NSs. Human normal prostatic myofibroblast (WPMY-1) cells were cultured in DMEM (Dulbecco's modified Eagle's medium, KeyGen, BioTech) with 10% fetal bovine serum (FBS, Gibco) medium at 37°C under 5% CO_2_. WPYM-1 cells were added in 96-well plates with the concentration of 10^4^ cell per well. After being cultured for 24 h, the supernatant medium was removed and MnO_2_ NS and MBP NS dispersions (200 *μ*L, suspended in DMEM (without FBS)) containing different concentrations of MnO_2_ (0, 20, 40, 80, 160, and 320 *μ*g/mL) were added into the 96-well plate. Cells cultured without any nanosheets were set as low control, and those cultured with 1% Triton X-100 were used as high control. After 24 h, 100 *μ*L of supernatant in each well was transferred into another 96-well plate, and 100 *μ*L reaction solution was added into the corresponding well. After 30 min, a microplate reader (PowerWave XS2, BioTek) was used to measure the optical density at 495 nm (OD_495_). The viability of cells was calculated according to the following formula

(3) $$\begin{array}{c} \text{Cell viability}=100\%-\left(A_{\text{T}}–A_{\text{L}}\right)/\left(A_{\text{H}}–A_{\text{L}}\right)\times 100\% \end{array}$$

where *A*_T_ represents the OD_495_ of test sample, *A*_L_ represents the OD_495_ of low control, and *A*_H_ represents the OD_495_ of high control.

### 4.8. Bacterial Culture

Methicillin-resistant Staphylococcus aureus (MRSA, ATCC43300) was used in this study. Before being used, a single colony of MRSA was transferred into 5 mL Luria-Bertani (LB) culture and cultured for 10 h. The bacterial dispersion was washed with saline and quantitated by a microplate reader. To obtain mature MRSA biofilms, MRSA dispersion in LB culture (containing 1% glucose, 10^7^ CFU/mL) was cultured at 37°C for 48 h.

### 4.9. Ce6 Release in MRSA Biofilms

For pH-responsive Ce6 release study, MRSA biofilms were cultured in a confocal petri dish. The MBP NSs (MnO_2_: 100 *μ*g/mL; Ce6: 80 *μ*g/mL) were cultured with MRSA biofilms at 37°C at different times (1, 4, 8, and 12 h). After being washed with saline for three times, MRSA biofilms were stained with Calcein-AM (KeyGen BioTech) for 30 min and then imaged by an Olympus IX81 confocal laser scanning microscope. The 3D images were obtained by using the FV10-ASW Viewer.

### 4.10. In Vitro Photodynamic Treatment of MRSA Biofilms

To treat the MRSA biofilms by PDT, MBP NSs (MnO_2_: 10, 50, and 100 *μ*g/mL) and MBP-Ce6 NSs (MnO_2_: 10, 50, and 100 *μ*g/mL and Ce6: 8, 40, and 80 *μ*g/mL) were added into MRSA biofilms with or without H_2_O_2_ (50 *μ*M). After being cultured for 12 h, the MRSA biofilms in the laser treatment groups were irradiated with 635 nm laser (20 mW/cm^2^). MRSA biofilms after different treatments were harvested by ultrasonication. The standard plate count method was used to determine the number of viable bacteria inside MRSA biofilms.

For fluorescence imaging, the treated MRSA biofilms were stained with Calcein-AM for 30 min and imaged by using a confocal laser scanning microscope (Olympus IX81). For crystal violet staining, MRSA biofilms were fixed with formalin for 10 min and dyed with crystal violet solution (0.02%) for 1 h. Images were captured by using a motorized fluorescence microscope (Olympus IX71). For SEM imaging, MRSA biofilms were grown on the surface of an indium tin oxide (ITO) glass. After treatment, MRSA biofilms were fixed in 2.5% glutaraldehyde solution for 30 min, dehydrated with series of ethanol solutions with gradient concentrations (15%, 30%, 50%, 75%, and 100%) for 15 min, respectively. After sputtering coated with gold, the morphology characterization of bacteria was performed on a Hitachi S4800 SEM.

### 4.11. MRSA Biofilm-Infected Animal Models

All animal procedures were performed in accordance with the Guidelines for Care and Use of Laboratory Animals, and experiments were approved by the Animal Ethics Committee of Nanjing University. Female Balb/c mice (20 g, 6 weeks old) were purchased from Nanjing Junke Biological Engineering Co., Ltd and used in this study. To develop the MRSA biofilm-infected mice model, MRSA suspensions in PBS (50 *μ*L, 10^9^ CFU/mL) were subcutaneously injected into the mice skin. After being infected for 2 days, subcutaneous abscesses appeared in the treated mice, indicating the formation of MRSA biofilms.

### 4.12. FL and MR Imaging of MRSA Biofilm-Infected Mice

Before the FL and MR imaging, MBP-Ce6 NSs dispersions were intravenously (i.v.) injected into MRSA-infected mice (dose of MnO_2_ = 5 mg/kg, dose of Ce6 = 4 mg/kg). Fluorescence images of mice at different times (1 h, 4 h, 8 h, 12 h, and 24 h) postinjection were captured on an IVIS Lumina K Series III system (Perkin Elmer). MR imaging was conducted on a MRI scanner system (BioSpec 7T/20 USR, Bruker) at different times (1 h, 4 h, 8 h, 12 h, and 24 h) postinjection of MBP-Ce6 NSs. The MRI images were obtained by using the Sante MRI Viewer.

### 4.13. Photodynamic Therapy of MRSA Biofilm-Infected Mice

Thirty mice with MRSA biofilm infections were randomly divided into five groups: (1) saline + laser, (2) MBP NSs + laser, (3) Ce6 + laser, (4) MBP-Ce6 NSs, and (5) MBP-Ce6 NSs + laser. The mice in groups 1-3 were i.v. injected with saline (200 *μ*L), MBP NS dispersion in PBS (200 *μ*L; MnO_2_: 5 mg/kg), Ce6 dispersion in PBS (200 *μ*L; dose of Ce6: 4 mg/kg), respectively. The mice in groups 4 and 5 were i.v. injected with MBP-Ce6 NSs dispersions (200 *μ*L; MnO_2_: 5 mg/kg, Ce6: 4 mg/kg). At 8th hour postinjection, the mice in the laser treatment groups (groups 1, 2, 3, and 5) were irradiated with 635 nm laser at the power density of 20 mW/cm^2^ for 30 min. The size of the infected tissues was measured by using caliper every 2 days. The area of the infected tissues was calculated according to the formula: area = (width/2 × length/2) × *π*. After the treatment for 8 days, the infected tissues of the mice were harvested and ultrasonicated for 10 min to disperse the bacteria. Then, the CFU numbers of the bacteria in the infected tissues were determined by the plate count method.

For histological analysis, the infected tissues of mice were harvested and fixed with 4% polyoxymethylene and then processed by standard protocols for hematoxylin and eosin (H&E) staining and Masson's trichrome staining, respectively.

### 4.14. Immunofluorescence Staining of MRSA Biofilm-Infected Tissues

MRSA biofilm-infected mice were i.v. injected with saline (200 *μ*L), Ce6 (200 *μ*L, 4 mg/kg), MBP NSs (200 *μ*L, MnO_2_: 5 mg/kg), and MBP-Ce6 NSs (200 *μ*L, MnO_2_: 5 mg/kg, Ce6: 4 mg/kg), respectively. At 8th hour postinjection, the infected tissues of mice were harvested, fixed with 4% polyoxymethylene, embedded in paraffin, and sectioned into slices. Triton X-100 solution (0.5%) was used for cell lysis. BSA was used to prevent nonspecific adsorption. The tissue slices were incubated with HIF-1*α* mouse monoclonal antibody at 4°C overnight. Then, the FITC-labeled goat anti-mouse secondary antibody was added at 37°C for 2 h. Cell nuclei were stained with 4′,6-diamidino-2-phenylindole (DAPI). Fluorescence images of the stained tissue slices were obtained by using the confocal laser scanning microscope (Olympus IX81).

### 4.15. Toxicity Study of MBP-Ce6 NSs In Vivo

Twelve Balb/c mice were randomly divided into two groups (6 mice per group) and were i.v. injected with 200 *μ*L PBS (group a) and 200 *μ*L MBP-Ce6 NSs (group b; MnO_2_: 10 mg/kg, Ce6: 8 mg/kg), respectively. At 28th day postinjection, one mouse was sacrificed to afford the major organs (heart, liver, spleen, lung, and kidney) for H&E staining. The blood of the other five mice was collected for hematology assay. For the blood chemistry analysis, the levels of alkaline phosphatase (ALP), alanine aminotransferase (ALT), aspartate aminotransferase (AST), and urea nitrogen (BUN) were measured. For the complete blood panel test, the levels of white blood cells (WBC), red blood cells (RBC), platelets (PLT), hemoglobin (Hgb), hematocrit (HCT), mean corpuscular volume (MCV), mean corpuscular hemoglobin concentration (MCHC), and mean corpuscular hemoglobin (MCH) were measured.

## Data Availability

All data needed to evaluate the conclusions in the paper are present in the paper. Additional data related to this paper may be requested from the authors.

## Supplementary Materials

Supplementary Materials Figure S1: TEM image of the as-prepared MnO_2_ NSs aggregations. Figure S2: large-scale TEM images and size statistics of MnO_2_ NSs, MBP NSs, and MBP-Ce6 NSs. Figure S3: selected area electron diffraction pattern and elemental mapping images of MnO_2_ NSs. Figure S4: large-scale AFM images and thickness statistics of MnO_2_ NSs, MBP NSs, and MBP-Ce6 NSs. Figure S5: hydrodynamic diameter and zeta potential of MnO_2_ NSs, MBP NSs, and MBP-Ce6 NSs. Figure S6: fluorescence recovery of MBP-Ce6 NSs at different conditions. Figure S7: the generation of singlet oxygen by MBP-Ce6 NSs. Figure S8: the colloidal stability characterization of MBP-Ce6 NSs. Figure S9: the cytotoxicity of MBP-Ce6 NSs. Figure S10: the ROS in MRSA biofilms treated by MBP-Ce6 NSs and H_2_O_2_. Figure S11: the crystal violet staining images of MRSA biofilms after photodynamic treatment. Figure S12: photographs of MRSA biofilm-infected tissues and nitrocellulose membrane contacted with the infected issues and followed by ruthenium red staining. Figure S13: the quantitative data of the FL and MRI imaging for the biofilm-infected mice. Figure S14: fluorescence images of the major organs and MRSA biofilm-infected tissues from the mice treated by MBP-Ce6 NSs. Figure S15: T_1_-weighted MR images of the mice in longitudinal section after i.v. injection of MBP-Ce6 NSs. Figure S16: biodistribution of Mn in major organs and MRSA biofilm-infected tissues from the mice treated by MBP-Ce6 NSs. Figure S17: analysis of H&E-stained slices of MRSA-infected tissues from mice. Figure S18: the *in vivo* toxicity evaluation of MBP-Ce6 NSs.
