# Supplementary material for: Biofilm Microenvironment-Responsive Nanotheranostics for Dual-Mode Imaging and Hypoxia-Relief-Enhanced Photodynamic Therapy of Bacterial Infections
Source: Research (Wash D C). 2020 Apr 12;2020:9426453. doi: 10.34133/2020/9426453 (PMC7128073; doi:10.34133/2020/9426453)
Supplement: Supplementary Materials — Figure S1: TEM image of the as-prepared MnO2 NSs aggregations. Figure S2: large-scale TEM images and size statistics of MnO2 NSs, MBP NSs, and MBP-Ce6 NSs. Figure S3: selected area electron diffraction pattern and elemental mapping images of MnO2 NSs. Figure S4: large-scale AFM images and thickness statistics of MnO2 NSs, MBP NSs, and MBP-Ce6 NSs. Figure S5: hydrodynamic diameter and zeta potential of MnO2 NSs, MBP NSs, and MBP-Ce6 NSs. Figure S6: fluorescence recovery of MBP-Ce6 NSs at different conditions. Figure S7: the generation of singlet oxygen by MBP-Ce6 NSs. Figure S8: the colloidal stability characterization of MBP-Ce6 NSs. Figure S9: the cytotoxicity of MBP-Ce6 NSs. Figure S10: the ROS in MRSA biofilms treated by MBP-Ce6 NSs and H2O2. Figure S11: the crystal violet staining images of MRSA biofilms after photodynamic treatment. Figure S12: photographs of MRSA biofilm-infected tissues and nitrocellulose membrane contacted with the infected issues and followed by ruthenium red staining. Figure S13: the quantitative data of the FL and MRI imaging for the biofilm-infected mice. Figure S14: fluorescence images of the major organs and MRSA biofilm-infected tissues from the mice treated by MBP-Ce6 NSs. Figure S15: T1-weighted MR images of the mice in longitudinal section after i.v. injection of MBP-Ce6 NSs. Figure S16: biodistribution of Mn in major organs and MRSA biofilm-infected tissues from the mice treated by MBP-Ce6 NSs. Figure S17: analysis of H&E-stained slices of MRSA-infected tissues from mice. Figure S18: the in vivo toxicity evaluation of MBP-Ce6 NSs. [file 9426453.f1.docx]

Supporting Information

**Biofilm Microenvironment-Responsive Nanotheranostics for Dual-mode Imaging and Hypoxia-Relief-Enhanced Photodynamic Therapy of Bacterial Infections**

Weijun Xiu^1^, Siyu Gan^1^, Qirui Wen^1^, Qiu Qiu^1^, Sulai Dai^1^, Heng Dong^4^, Qiang Li^4^, Lihui Yuwen^1,^*, Lixing Weng^2^, Zhaogang Teng^3^, Yongbin Mou^4^, and Lianhui Wang^1,^*

*^1^Key Laboratory for Organic Electronics and Information Displays & Jiangsu Key Laboratory for Biosensors, Institute of Advanced Materials (IAM), Jiangsu National Synergetic Innovation Centre for Advanced Materials (SICAM), Nanjing University of Posts and Telecommunications, Nanjing 210023, China*

*^2^School of Geography and Biological Information, Nanjing University of Posts and Telecommunications, Nanjing, 210023, China*

*^3^Department of Medical Imaging, Jinling Hospital, School of Medicine, Nanjing University, Nanjing, 210002, China*

*^4^Department of Oral Implantology, Nanjing Stomatological Hospital, School of Medicine, Nanjing University, Nanjing, 210023, China*

^*^Correspondence should be addressed to Lihui Yuwen; [iamlhyuwen@njupt.edu.cn](mailto:iamlhyuwen@njupt.edu.cn) and Lianhui Wang; iamlhwang@njupt.edu.cn


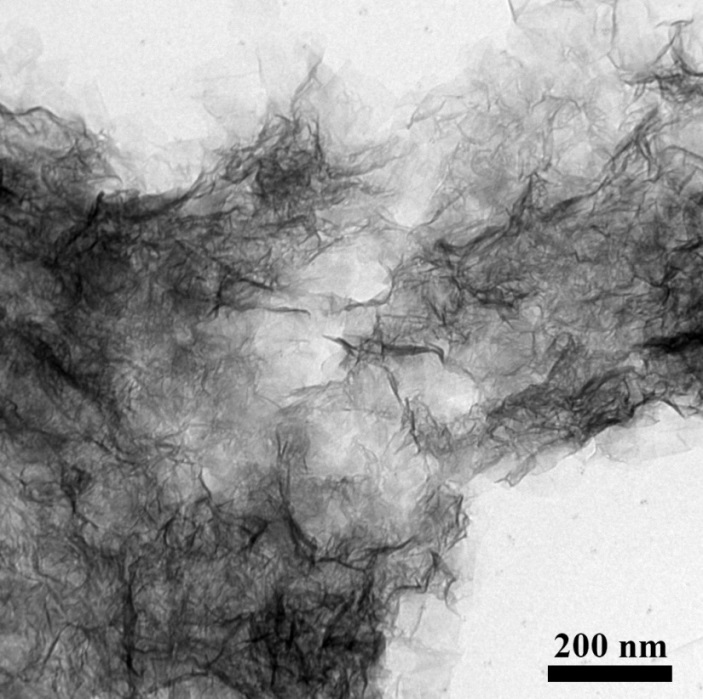


Figure S1 TEM image of the as-prepared MnO_2_ NSs aggregations.


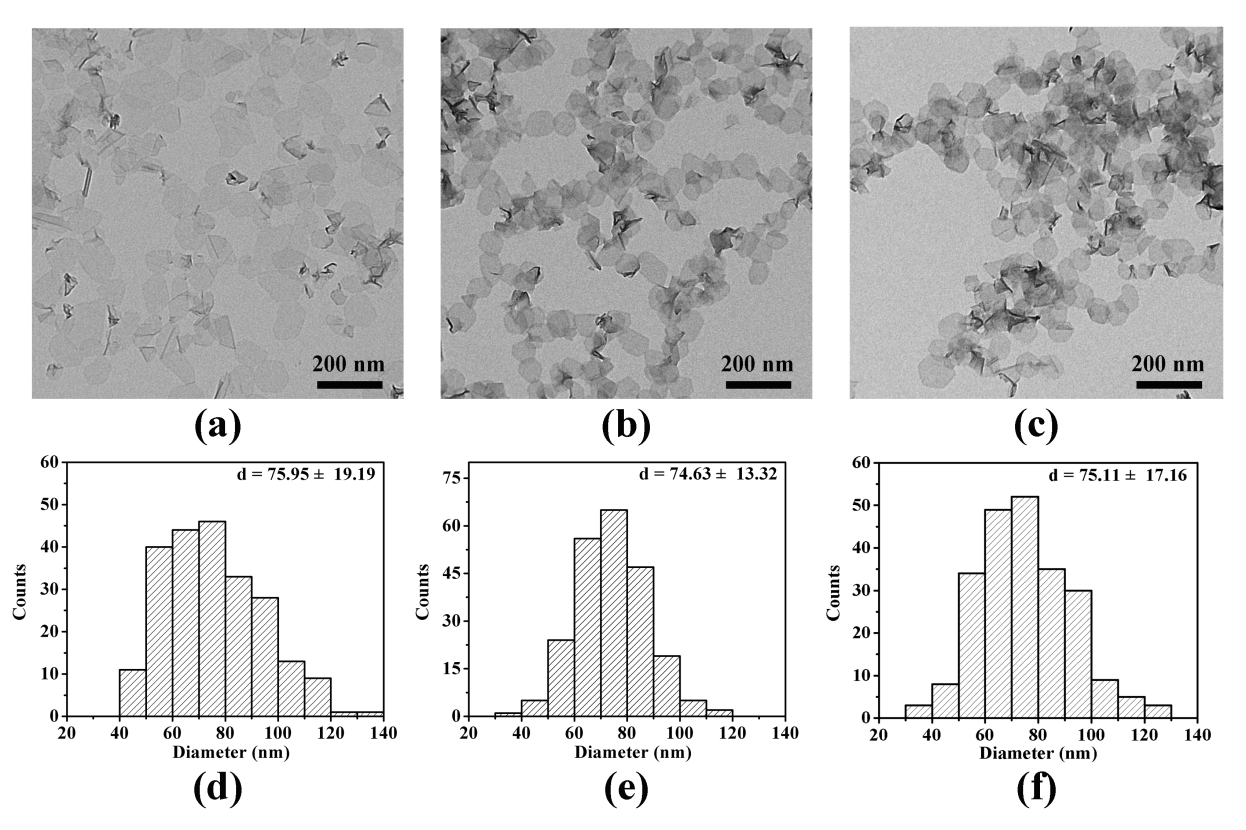


Figure S2 TEM images of (a) MnO_2_ NSs, (b) MBP NSs, and (c) MBP-Ce6 NSs. Size statistics of (d) MnO_2_ NSs, (e) MBP NSs, and (f) MBP-Ce6 NSs. Data are based on more than 200 nanosheets and presented as mean ± s.d.


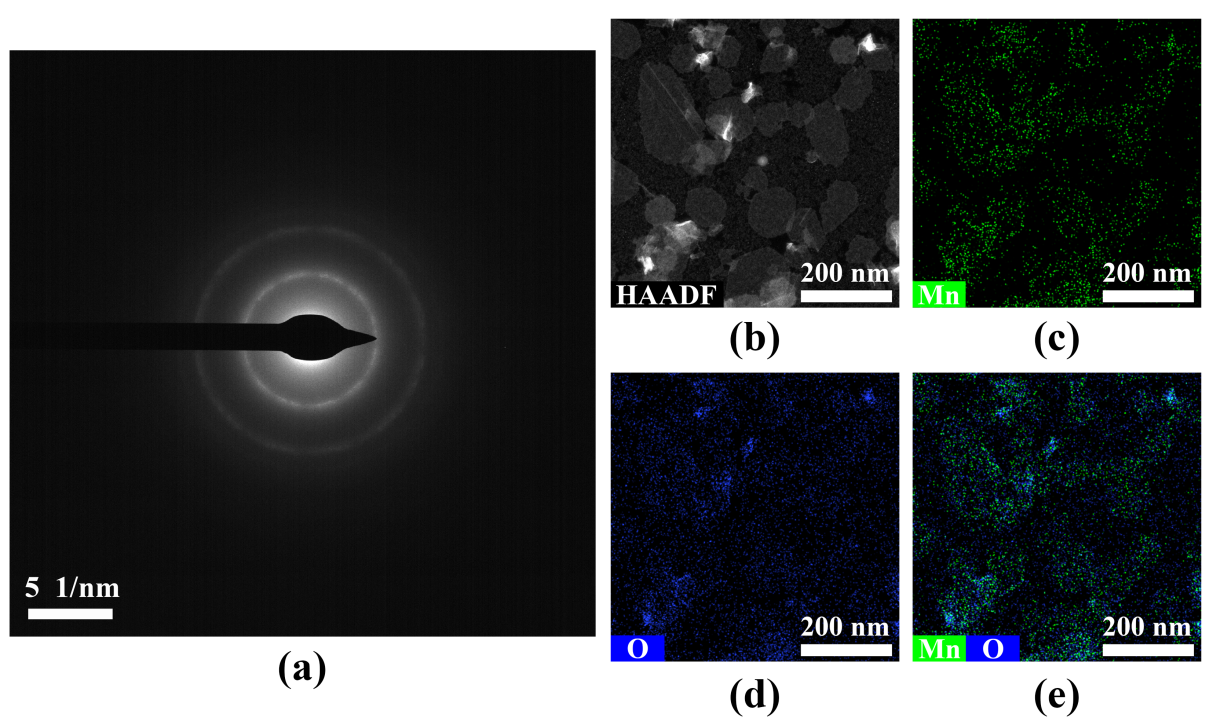


Figure S3 (a) Selected area electron diffraction (SAED) pattern, (b) High-angle annular dark field-scanning transmission electron microscopy (HAADF-STEM), and (c-e) energy dispersive spectroscopy (EDS) elemental mapping images of MnO_2_ NSs.


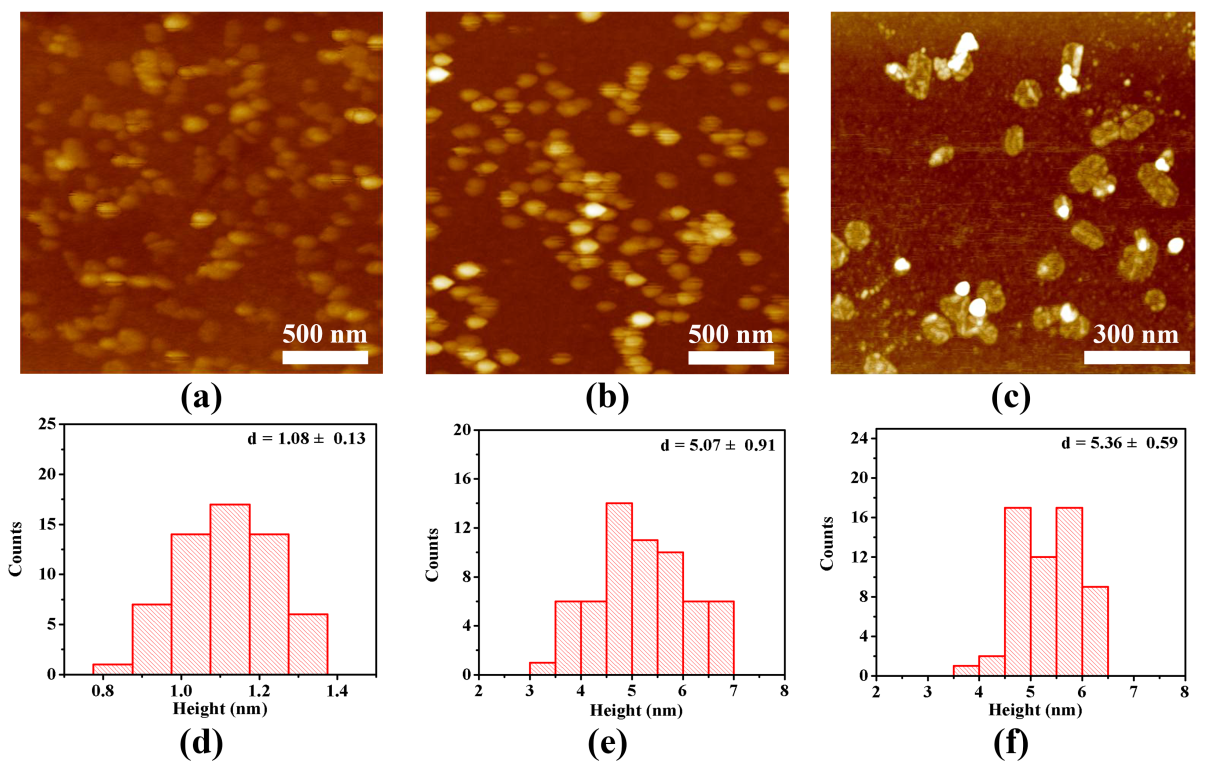


Figure S4 AFM images of (a) MnO_2_ NSs, (b) MBP NSs, and (c) MBP-Ce6 NSs. Thickness statistics of (c) MnO_2_ NSs, (d) MBP NSs, and (e) MBP-Ce6 NSs. Data are based on more than 50 nanosheets and presented as mean ± s.d.


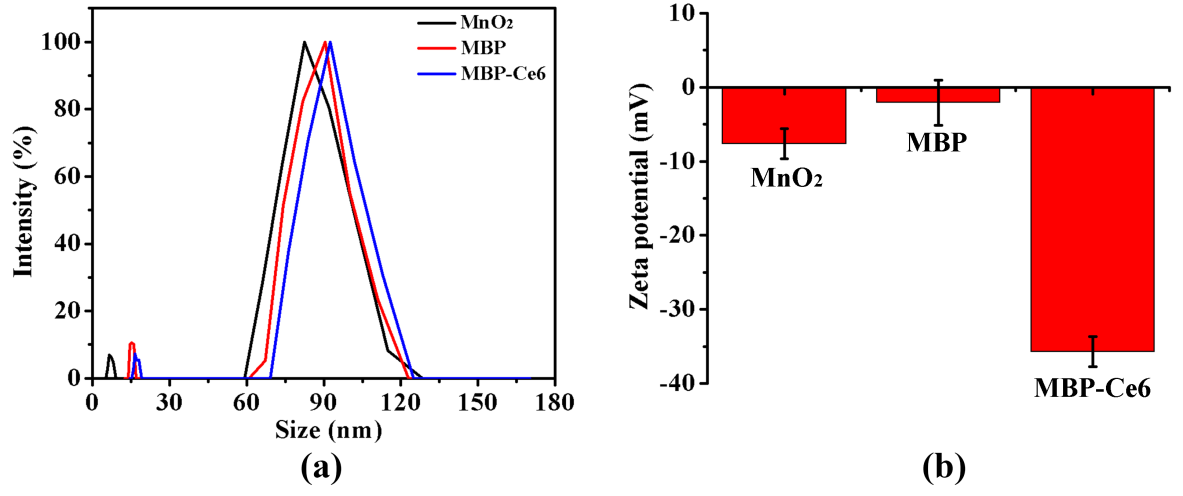


Figure S5 (a) Hydrodynamic diameter and (b) Zeta potential of MnO_2_ NSs, MBP NSs, and MBP-Ce6 NSs. Error bars indicate s.d. (n=3).





Figure S6 Fluorescence recovery of MBP-Ce6 NSs (Ce6: 20 μg/mL) in PBS with different pH values (7.4, 6.0, and 5.0) at different time points.


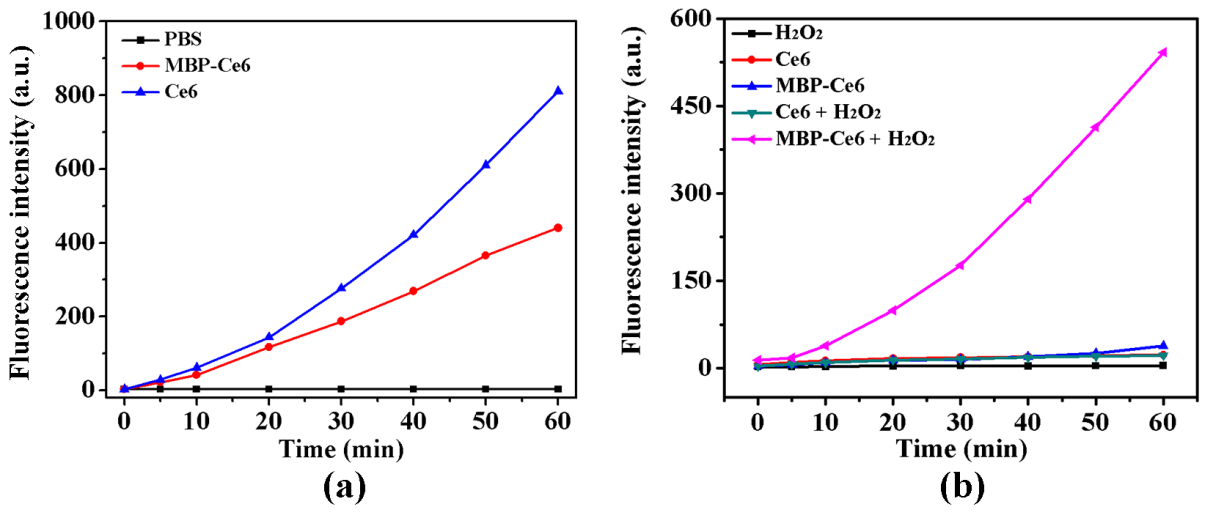


Figure S7 The study of singlet oxygen generation of MPB-Ce6 in normoxic (a) and hypoxic (b) conditions by using DCFH-DA as a fluorescence sensor. In normoxic conditions，Ce6 (10 μg/mL) and MBP-Ce6 NSs (MnO_2_: 12.5 μg/mL; Ce6: 10 μg/mL) were dispersed in PSB (pH=7.4). In hypoxic conditions, all solutions, including H_2_O_2_ (50 μM), Ce6 (10 μg/mL), MBP-Ce6 NSs (MnO_2_: 12.5 μg/mL; Ce6: 10 μg/mL), Ce6 + H_2_O_2_ (Ce6: 10 μg/mL; H_2_O_2_: 50 μM), and MBP-Ce6 NSs+ H_2_O_2_ (MnO_2_: 12.5 μg/mL; Ce6: 10 μg/mL; H_2_O_2_: 50 μM), were saturated by nitrogen and then irradiated by 635 nm laser for 60 min at the power density of 20 mW/cm^2^.


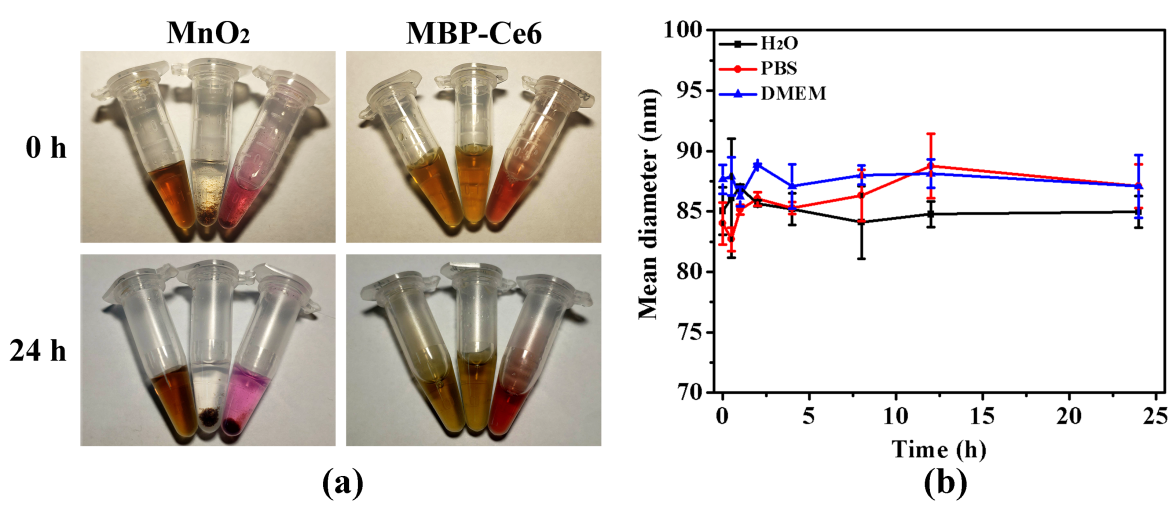


Figure S8 (a) The photographs of MnO_2_ NS (50 μg/mL) and MBP-Ce6 NSs (MnO_2_: 50 μg/mL; Ce6: 40 μg/mL) dispersed in H_2_O, PBS, and DMEM at different time points. (b) The change of mean hydrodynamic diameter of MBP-Ce6 NSs after dispersed in H_2_O, PBS, and DMEM for different times. Error bars indicate s.d. (n=3).


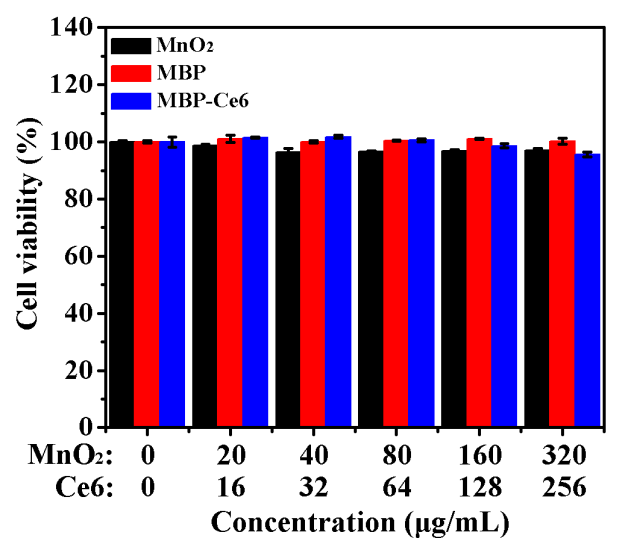


Figure S9 Cells viability of WPMY-1 after cultured with MBP-Ce6 NSs at different concentrations (MnO_2_: 0, 20, 40, 80, 160, and 320 μg/mL; Ce6: 0, 16, 32, 64, 128, and 256 μg/mL) for 24 h. Error bars indicate s.d. (n=3).


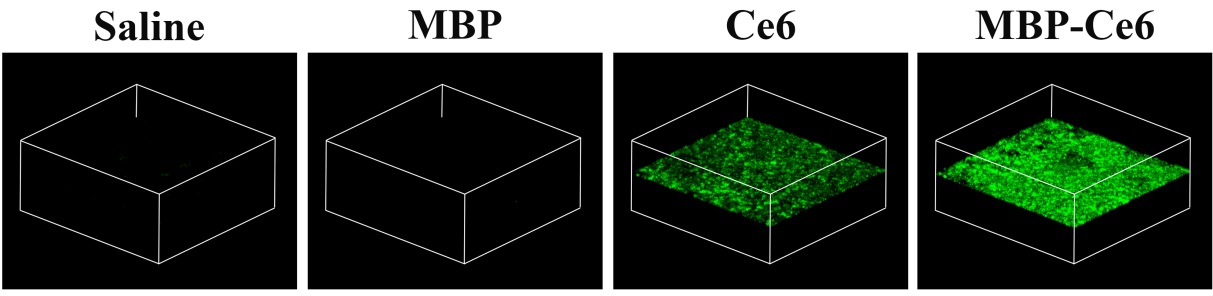


Figure S10 Fluorescence images of the MRSA biofilms after different treatments (saline, MBP NSs (MnO_2_ NSs: 100 μg/mL), Ce6 (80 μg/mL), and MBP-Ce6 NSs (MnO_2_: 100 μg/mL; Ce6: 80 μg/mL)) with the presence of H_2_O_2_ (50 μM) and followed by laser irradiation (635 nm, 20 mW/cm^2^, 30 min) and DCFH-DA staining. The size of the CLSM images is 630 μm × 630 μm.


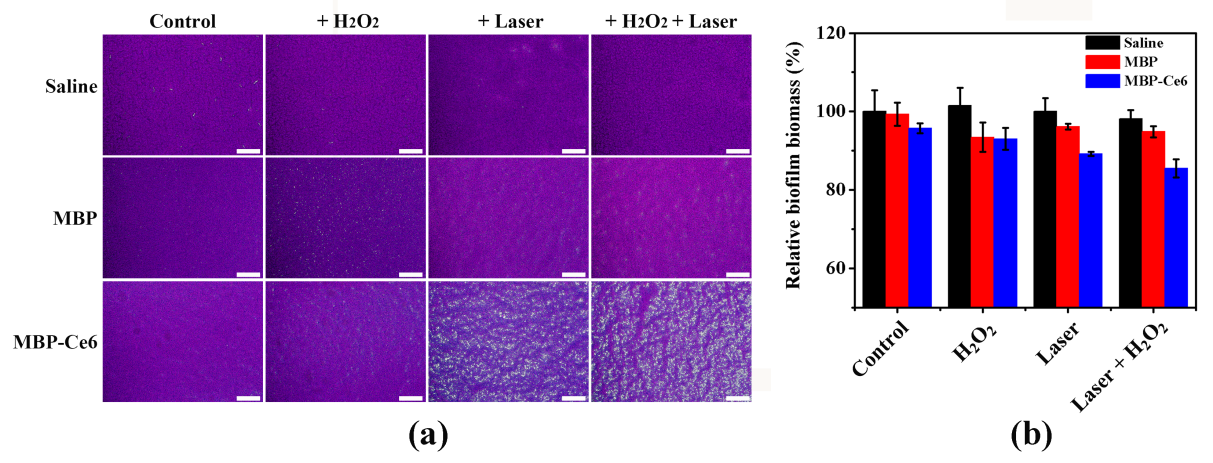


Figure S11 (a) Micrographs of the MRSA biofilms treated by saline, MBP NSs (MnO_2_ NSs: 100 μg/mL), and MBP-Ce6 NSs (MnO_2_: 100 μg/mL; Ce6: 80 μg/mL) at different conditions and followed by crystal violet staining. (b) Semi-quantitative analysis of the relative biofilm biomass in different groups shown in (a). The concentration of H_2_O_2_ is 50 μM. The laser irradiation was performed at the power density of 20 mW/cm^2^ (635 nm) for 30 min. The scale bar is 100 μm. Error bars indicate s.d. (n=3).


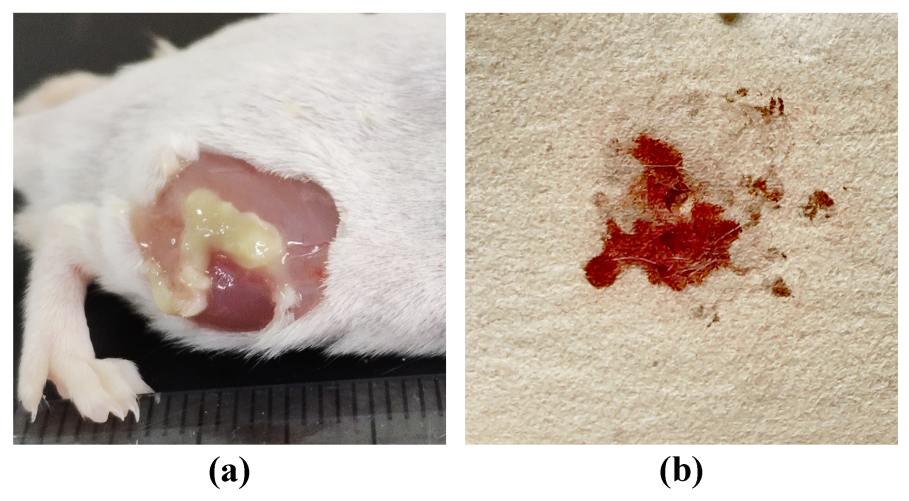


Figure S12 (a) Photograph of MRSA biofilm infected tissues in the mice used in this study. (b) Photograph of the ruthenium red stained nitrocellulose membrane after applied to the MRSA biofilm infected tissues.


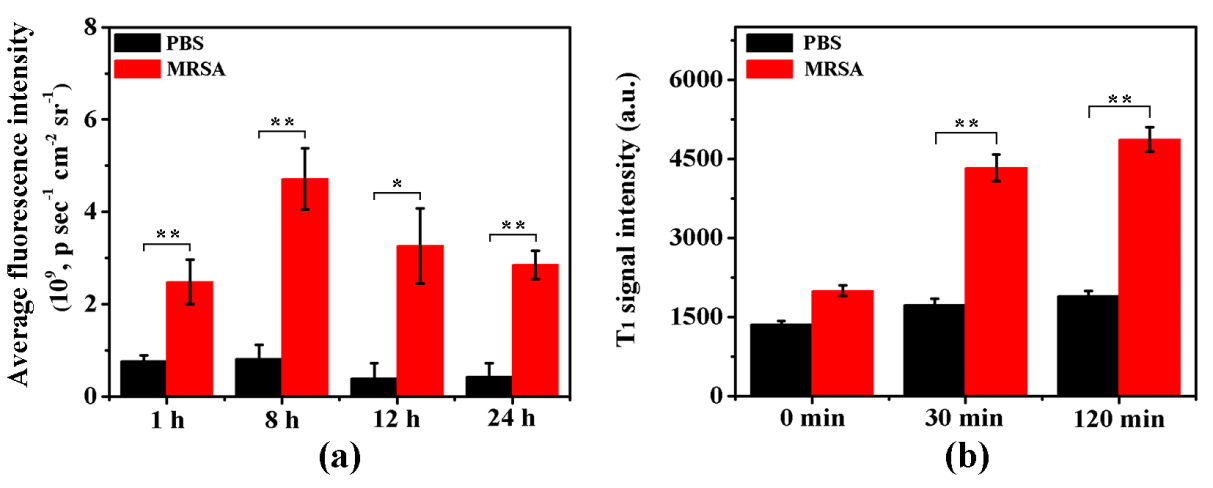


Figure S13 (a) The average fluorescence intensity and (b) T_1_-weighted MR signal of different tissues after *in situ* injection of PBS and MBP-Ce6 NSs in PBS (MnO_2_: 50 μg/mL; Ce6: 40 μg/mL) for different times. **p* < 0.05, ***p* < 0.01 (two-tailed Student’s *t*-test).


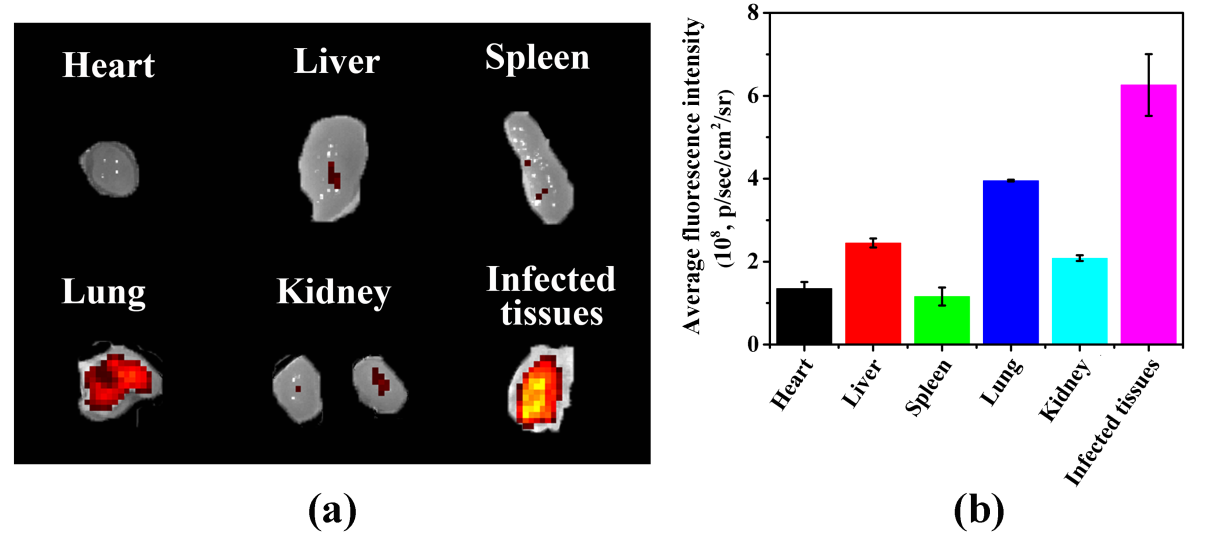


Figure S14 (a) Fluorescence images of the major organs (heart, liver, spleen, lung and kidney) and biofilm infected tissues from the mice after i.v. injected with MBP-Ce6 NSs (MnO_2_: 5 mg/kg; Ce6: 4 mg/kg) for 24 h, and (b) their average fluorescence intensity. Data are presented as means ± s.d. (n = 3).


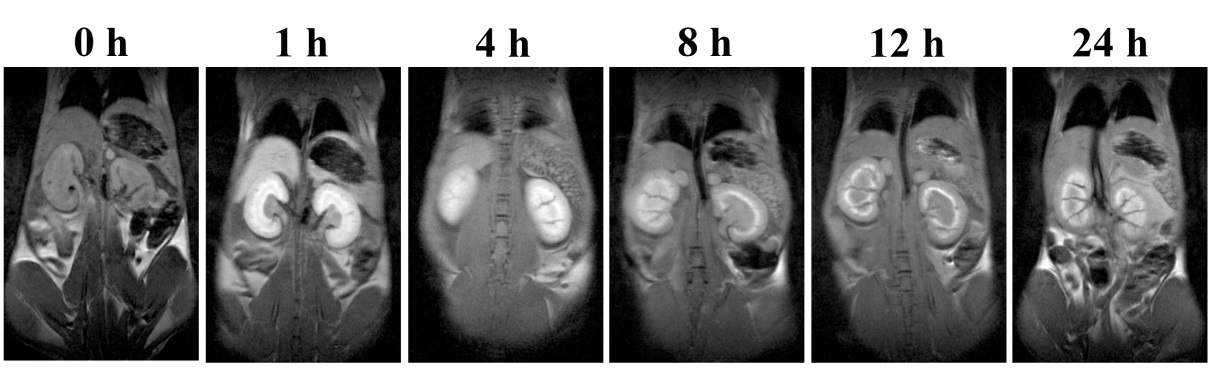


Figure S15 T_1_-weighted MR images of the mice in longitudinal section after i.v. injection of MBP-Ce6 NSs (MnO_2_: 5 mg/kg; Ce6: 4 mg/kg) for different times.


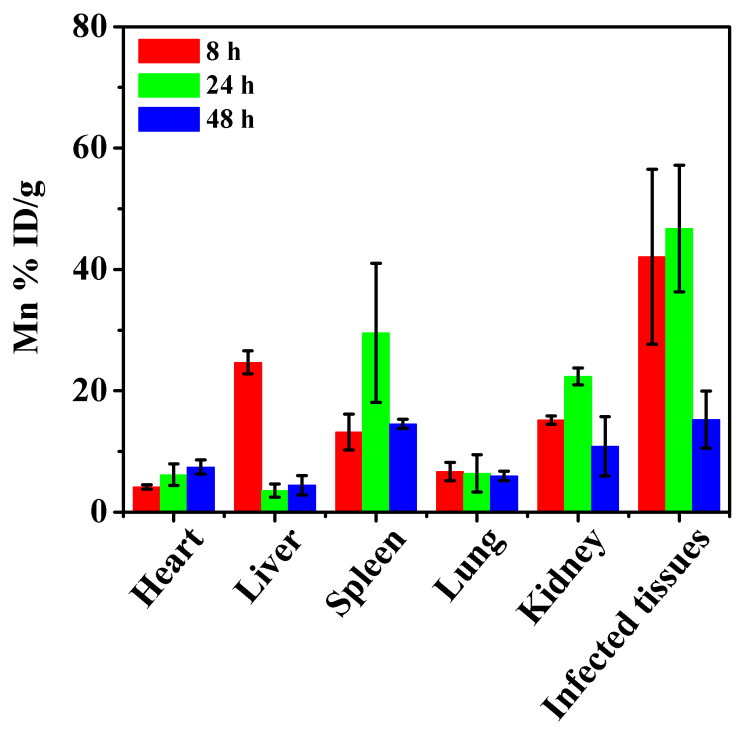


Figure S16 Biodistribution of Mn in major organs (heart, liver, spleen, lung, and kidney) and MRSA biofilm infected tissues at different times post-injection. The quantitation of Mn were performed by using ICP-AES. Data are presented as means ± s.d. (n = 3).


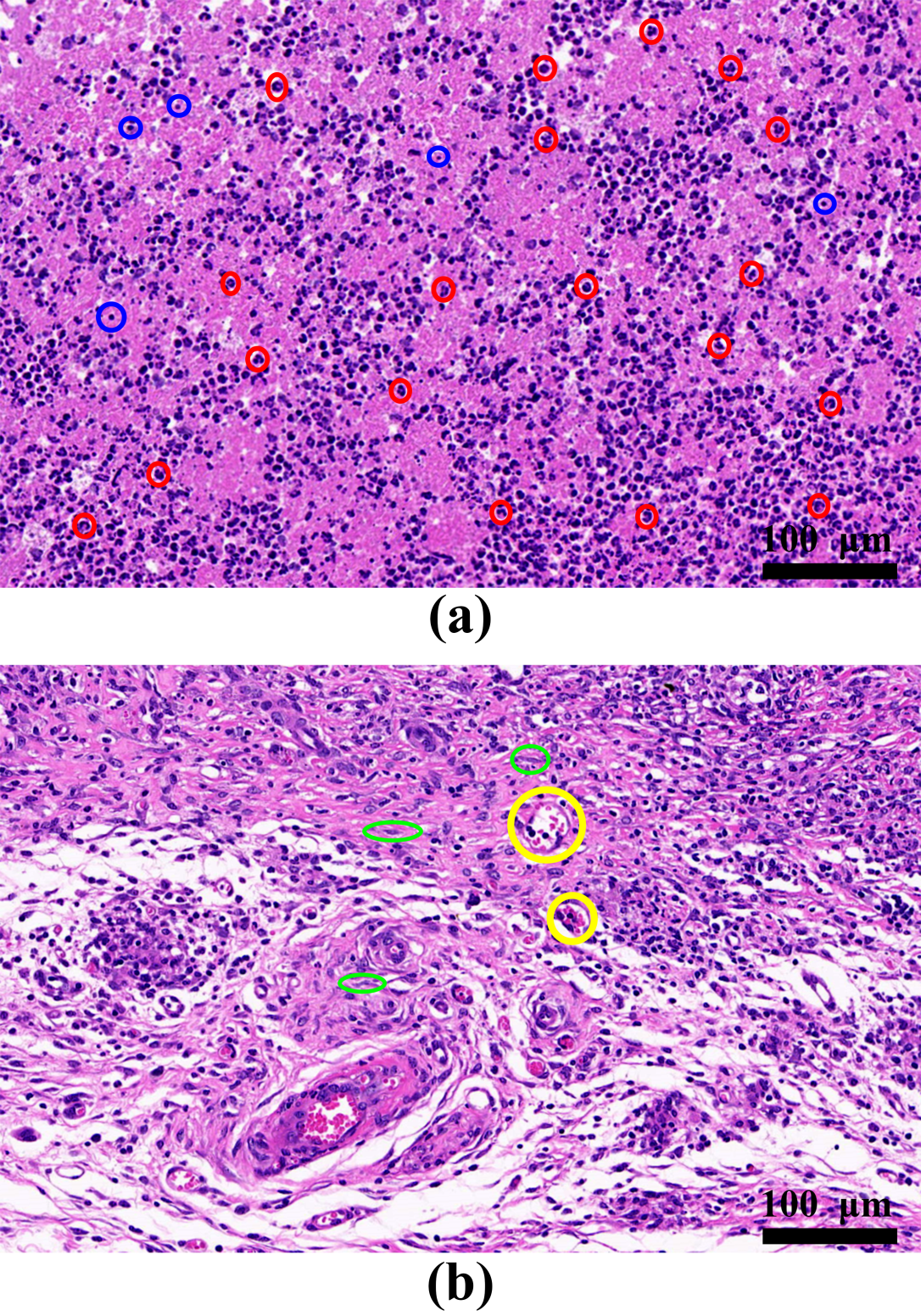


Figure S17 Enlarged hematoxylin and eosin (H&E) staining images of the biofilm infected tissue slices from the mice treated by (a) laser irradiation only and (b) MBP-Ce6 NSs with laser irradiation. Red, blue, and green circles indicate the neutrophil granulocytes, lymphocytes, and fibroblasts, respectively. Yellow circles denote neovascularization.


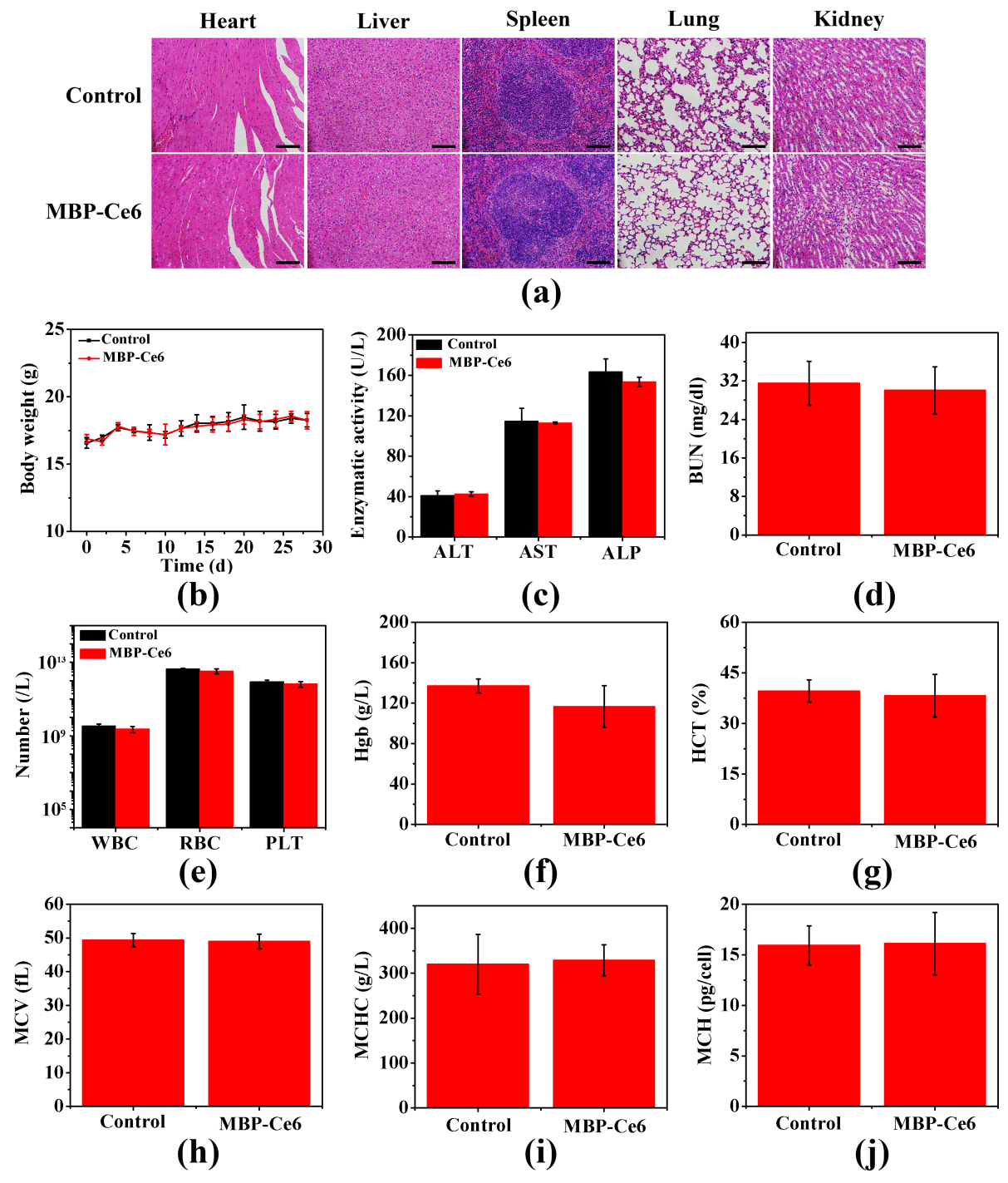


Figure S18 (a) H&E stained slices of major organs from the mice treated with PBS (control) and MBP-Ce6 NSs at 28 d. The scale bar is 100 μm. (b) The body weight curves of the mice after i.v. injection with PBS and MBP-Ce6 NSs. (c) Alanine aminotransferase (ALT), aspartate aminotransferase (AST), alkaline phosphatase (ALP), (d) blood urea nitrogen (BUN), (e) white blood cells (WBC), red blood cells (RBC), platelets (PLT), (f) hemoglobin (Hgb), (g) hematocrit (HCT), (h) mean corpuscular volume (MCV), (i) mean corpuscular hemoglobin concentration (MCHC), and (j) mean corpuscular hemoglobin (MCH) levels in the blood at 28 d post-injection of PBS (control) or MBP-Ce6 NSs. All data are presented as means ± s.d. (n = 5).
